# Assessing Sub-Saharan Africa’s readiness to address the impact of climate change and health: A scoping review

**DOI:** 10.1371/journal.pone.0315482

**Published:** 2025-11-11

**Authors:** Aminata Kilungo, God’sgift Chukwuonye, Victor Okpanachi, Hussein Mohamed

**Affiliations:** 1 Department of Community, Environment and Policy, University of Arizona, Tucson, Arizona, United States of America; 2 Department of Environmental Sciences, The University of Arizona, Tucson, Arizona, United States of America; 3 Department of Environmental and Occupational Health, Muhimbili University of Health and Allied Sciences, Dar es Salaam, Tanzania; Clinton Health Access Initiative, UNITED STATES OF AMERICA

## Abstract

Climate change severely threatens global public health, with sub-Saharan Africa (SSA) projected to experience profound impacts. This scoping review aimed to provide a comprehensive overview of current research on climate change and its health implications in SSA while identifying research gaps and outlining the necessary resources and policy interventions to strengthen public health resilience in the region. Literature was retrieved from four databases (PubMed, Scopus, Embase and Web of Science) using the keywords “climate change,” “health,” and “sub-Saharan Africa” and this study was conducted using the PRISMA framework. The inclusion criteria were peer-reviewed studies published in English between January 1, 2001, and August 1, 2024, that examined the effects of climate change in SSA, assessed its impacts on health outcomes,A total of 7851 journal articles were identified from the initial search, and after screening, 153 studies were included for review. The included studies were published between January 2001 and August 2024. Although extensive studies have been conducted on extreme heat (71 studies), drought (45 studies), extreme precipitation events (52 studies), and flooding (34 studies), important themes such as air quality (10 studies), chemical water quality (8 studies) and natural disasters (8 studies) have been understudied. Additionally, this scoping review revealed a geographical gap in climate change and health studies, as only 24 out of 53 countries in sub-Saharan Africa were represented. The key deficiencies identified include limited funding, technological constraints, inadequate climate policies, and a lack of community-focused adaptation plans. Moreover, this review highlights the urgent need for resilient healthcare systems capable of addressing climate-related health risks effectively. Addressing these gaps is essential for developing targeted strategies to mitigate climate change’s health impacts and increase resilience in SSA communities. This review aims to inform policymakers, researchers, and stakeholders about critical areas requiring attention and investment by enhancing our understanding of these challenges and gaps. Strengthening research capacities, fostering collaboration, and implementing evidence-based policies are imperative steps toward achieving sustainable health outcomes in the face of a changing climate in SSA.

## Introduction

Climate change poses a significant and escalating threat to public health globally, with particularly profound implications for vulnerable regions such as sub-Saharan Africa (SSA) and other low-income countries. Characterized by diverse climatic conditions and socioeconomic challenges, SSA faces heightened susceptibility to the multifaceted impacts of climate change [1–7]. Extreme heatwaves, shifting precipitation patterns, increased flooding frequency and intensity, and prolonged droughts directly influence health outcomes in the region [8–11]. These environmental shifts exacerbate existing health disparities and introduce new risks, impacting the well-being of more than one billion people [12] in SSA.

In SSA and other low-income countries, the impact of climate change will be more dire due to unaddressed structural and socioeconomic vulnerabilities, poor governance, and inadequate supportive policies to address climate change and health issues. These unaddressed issues have resulted in other challenges, including water insecurity, poor healthcare infrastructure, a lag in research and training needed to address climate issues, and others [13–15]. For instance, in places with a high burden of diarrhea disease due to a lack of Water, Sanitation, and Hygiene (WASH) services, climate change poses additional risks, as countries are now facing more diarrhea diseases and, in some areas, cholera outbreaks, most of which are attributed to climate change [2,16–20]. Health conditions that are expected to be exacerbated in addition to waterborne and water-related diseases include foodborne diseases, vectorborne diseases, mental health, high morbidity and mortality due to heat stroke, malnutrition, and many others associated with natural disasters. In these countries, we are already seeing overwhelmed healthcare systems that are not equipped to address these public health challenges [14,21,22].

Although some African countries have developed plans for adaptation and mitigation strategies as required by the UN [23], there is little evidence on whether and how these plans are implemented. In addition, limited research has been conducted in SSA to guide country-specific plans for adaptation and mitigation and other associated measures to address or reduce the impact of climate change on health [24,25]. Limited data is collected at the ground level to guide some of these plans and interventions. Most plans and research approaches are based on broader global perspectives [7,26–28]. Given that SSA has unique challenges, research to guide adaptation, mitigation, and resilience efforts must be unique and specific to the region, if not country-specific. Given these challenges, SSA needs to scale up research, education, climate change financing, and policy – all collectively, to move forward toward sustainable solutions for long-term climate change resilience. Most importantly, there is a need to set a road map for priorities to guide efforts, focusing on addressing challenges specific to the region.

To understand the research that has been conducted to guide some of these efforts, this scoping review, following the PRISMA framework, was conducted to examine current research on climate change and health impacts, policy, and to identify existing gaps in research and the need for policy-making to guide interventions to improve public health and climate change resilience in Africa. This scoping review aims to improve our understanding of where SSA is conducting research to generate specific data to address the complex challenges related to health outcomes due to climate change. The specific research questions guiding this review are as follows:

1. What are the geographic and thematic gaps in SSA’s climate change and health research, and how do these gaps affect our understanding of the region’s climate-induced health outcomes and vulnerabilities?
2. How do extreme weather events, such as heatwaves, droughts, and floods, interact with social determinants of health to influence health vulnerabilities and outcomes?
3. What practical solutions and community-based adaptation strategies can be developed and implemented to enhance resilience and mitigating health impacts to climate change
4. How are climate change and health studies funded?

## Method

**Fig 1:**
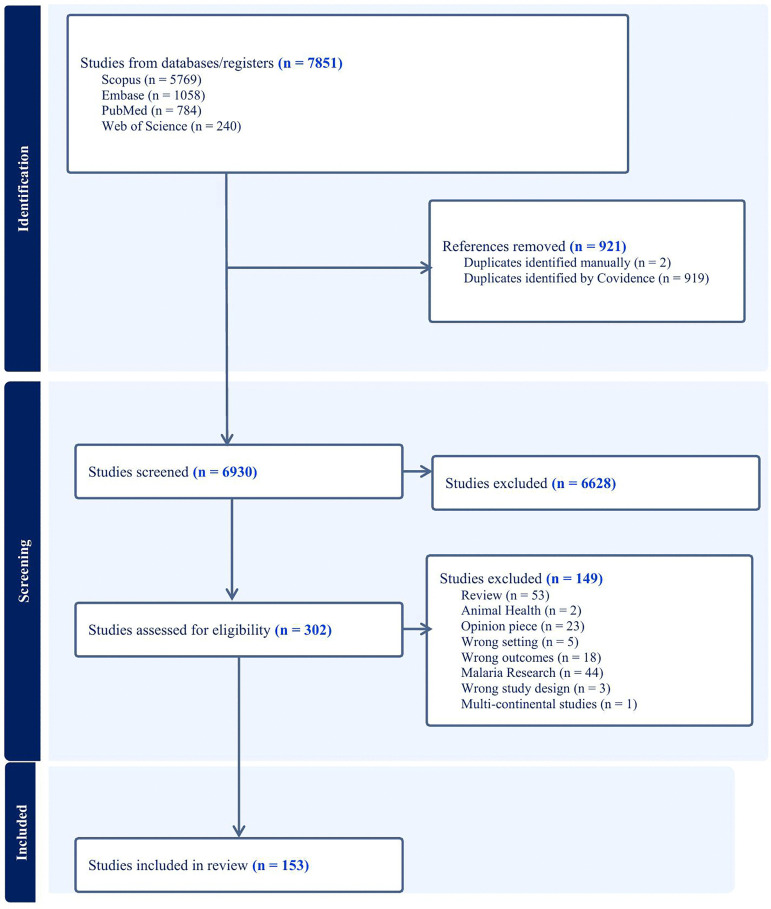
PRISMA flowchart showing the number of articles identified in PubMed, screened and included in the scoping review.

A scoping review was conducted on PubMed, Embase, Web of Science and Scopus using the keywords “climate change,” “health,” and “sub-Saharan Africa” and imported to Covidence [29] to streamline and facilitate the review. The Covidence software follows the PRISMA approach for scoping review (Fig 1). A total of 7851 journal articles were identified using the keywords and inclusion criteria. No white papers, gray literature, review papers, or other non-peer reviewed sources were included. Only peer-reviewed primary research articles published in English were considered. Although significant contributions to climate change knowledge particularly from community organizations, governments, NGOs, and interdisciplinary academic efforts are often published in gray literature and review formats, these sources were excluded to maintain methodological consistency, ensure reproducibility, and focus on studies presenting original empirical data. Additionally, such documents are often less accessible, not consistently archived, and may be less likely to inform formal planning or decision-making processes, which tend to rely more heavily on peer-reviewed literature.

Papers were included if they (a) explored the effects of climate change in sub-Saharan Africa, (b) explored the impacts of climate change on health outcomes, (c) focused on relevant climate change impacts, including water quality, flooding, and drought and other health outcomes (d) were published between January 1, 2001, and August 1, 2024, and (d) were written in English. 2001 was selected as the starting point because it marks the beginning of the third assessment cycle of the Intergovernmental Panel on Climate Change (IPCC) [30], which significantly advanced the global discourse on climate change and its regional impacts. This period also aligns with a noticeable increase in peer-reviewed literature focused on climate-health linkages in SSA, making it a logical starting point for capturing the evolution of research in this field.

On the other hand, papers were excluded if they were not from the sub-Saharan Africa region or if they mentioned climate change impacts not relevant to the African context (e.g., changes in snowmelt, sea ice loss or glacial retreat). Of the 7851 identified studies, 921 were duplicates, and 6628 were excluded because they did not meet the inclusion criteria. A total of 302 studies were screened for eligibility. Of the 302 studies, 149 were excluded; 53 were reviews, 2 were animal health research, 44 were malaria research, 23 were opinion pieces and correspondences, and 27 studies had the wrong geographical settings, study design or outcomes. In total, 153 studies were included for review (Fig 1). Malaria-focused studies were excluded due to the large existing body of literature examining the relationship between climate change and malaria in SSA. Therefore, this effort was directed toward other health outcomes that have received comparatively less attention in the literature. To reduce selection bias, study screening was conducted independently by two reviewers.

### Data extraction

Data extraction was conducted independently by two reviewers following the screening process. Each reviewer extracted key details from the included studies, including study characteristics (e.g., study design, population, country), climate change impacts discussed, health outcomes explored, and the types of interventions or solutions presented. Data were extracted into a structured data charting form within the Covidence platform to ensure consistency and minimize errors. Following the extraction process, a verification step was conducted to ensure that all relevant data had been captured accurately and consistently. This step included comparing the data extractions of both reviewers for discrepancies. Any inconsistencies were discussed and resolved through consensus, with a third reviewer consulted if necessary.

### Data analysis

The extracted data were stored securely in Covidence and later exported into an spreadsheet for analysis. A hybrid thematic analysis was employed to synthesize findings from the included studies. An initial set of themes was developed a priori, informed by existing literature on climate change and health in sub-Saharan Africa, as well as the objectives of this scoping review. These preliminary themes included: highlighting research needs, informing science, providing solutions and knowledge to combat climate impacts, highlighting policy needs, and identifying resource needs. During the data charting process, these themes were iteratively refined through an inductive review of the literature to capture additional patterns and insights that emerged across the studies. This combination of deductive and inductive thematic analysis ensured a structured yet flexible synthesis of findings. To verify the robustness of the thematic analysis, two independent researchers cross-checked the categorization of studies into themes. Any discrepancies in theme assignment were discussed and resolved through consensus.

Additionally, the countries where previous climate change and health studies were conducted were visualized on a heatmap, providing insight into which countries have been studied extensively and where significant gaps in information exist. The thematic groupings enabled a comprehensive understanding of how climate change affects the region and its inhabitants. By categorizing the literature in this way, we could pinpoint specific gaps in research and policy that need to be addressed. The map further highlighted underresearched regions, emphasizing the need for more focused studies in those areas.

Finally, results were visualized in tables, charts, figures, and maps, enabling a clear presentation of findings. The analysis helped identify practical solutions and resources that can be mobilized to mitigate the adverse effects of climate change on health outcomes in SSA. This approach ensures that the findings contribute to academic knowledge and provide actionable insights for policymakers, researchers, and practitioners working to combat climate change and its impacts on public health.

## Results

The majority of the studies published on climate change and health in sub-Saharan Africa between 2001 and August 2024 focused on extreme heat (71 studies), extreme precipitation events (52 studies), drought (45 studies), and flooding (34 studies). Additionally, a moderate number of studies have focused on infectious diseases (24 studies) and microbial water quality (23 studies). For almost 24 years, only 10 studies have been published on climate change and air quality and 8 studies on chemical water quality and natural disasters (Table 1).

**Table 1:** Climate Change Impacts in sub-Saharan Africa Identified by Past Studies.

Climate Impact	n	Studies
Extreme Heat	71^*^	[4,10,11,14,18,19,31–95]
Extreme Precipitation	52	[4,14,18,19,32–36,38–41,46–50,52,54,57,64,68,69,77–79,81,83,86,90,91,93–112]
Drought	45	[2,14,34,36–49,97–103,113–132]
Flooding	34	[2,4,8,14,19,35,38,40–48,52,56,57,67,68,90,95,97,99–101,103,107,128,133–136]
Microbial Water Quality	23	[20,35,61,81,95,105,106,108,135,137–151]
Infectious Diseases	23	[1,11,14,47,60,61,91,102,108,138,143,151–161]
Air Quality	10	[31–35,96,113,162–164]
Chemical Water Quality	8	[35,137,139,141,142,145,146,165]
Natural Disasters	8	[8,16,41,42,47,67,94,100]

* Some studies covered multiple themes. Hence, N ≠ 153.

Of the 153 studies reviewed, 58 informed science by providing insight into climate trends and impacts; 43 studies discussed specific solutions and knowledge to help mitigate the impact of climate change on health and resource needs, 20 of the studies discussed and highlighted research needs, and 15 studies highlighted policy. Additionally, 11 studies explored multiple themes (Table 2).

**Table 2:** Research Categories and Number of Studies in Identified Categories from Previous Studies in Sub-Saharan Africa.

	n	References
Informs science – Provides insight for climate trends and impacts	58	[1,4,8,11,33,34,36,39,42,46,67,68,71,73,74,77–85,88–91,93,102,106–108,110–113,120,129,130,132,133,136,143,145–147,149–151,155,159–161,164,166,167]
Provides solution and knowledge to combat climate impacts	43	[16,35,43,49,50,53,54,56,66,69,76,86,94,99–101,105,115–117,119,125–127,138–142,144,148,152,154,157,162,168–170]
Highlight research needs	20	[19,44,45,55,57,59,60,63–65,72,75,97,104,109,122,124,127,128,163]
Highlights policy needs	15	[14,38,40,41,62,70,87,92,96,134,153,156,171]
Highlights resources needs	*6*	[48,52,95,114,123,172]
Multiple Categories in a single study		
Highlight research needs; Highlights policy needs	3	[31,61,158]
Informs science – Provides insight for climate trends and impacts; Highlights resources needs	2	[98,103]
Informs science – Provides insight for climate trends and impacts; Highlights policy	2	[47,131]
Provides solution and knowledge to combat climate impacts; Highlights policy needs	1	[2]
Provides solution and knowledge to combat climate impacts; Highlights policy needs; Highlights resources needs	1	[137]
Provides solution and knowledge to combat climate impacts; Informs the state of climate science in Africa	2	[32,118]
Highlights policy needs: Highlights resources needs	1	[20]

Most studies included in this scoping review focused on water, sanitation and hygiene issues (n = 57), food security and malnutrition (n = 40), physical illness (n = 32) and health risks associated with pathogens (n = 26). The remaining 53 articles covered loss of livelihood due to natural disasters, climate induced displacement, mental health, gender-based violence, HIV and death (Table 3).

**Table 3:** Climate Change and Health Issues in sub-Saharan Africa Identified by Past Studies.

Climate Change Impact	N^*^	Studies
WASH issues	57	[1,2,11,14,16,18–20,33,34,36,38,43,44,52,56,66,81,86,91,93,94,101,104–109,113,115,122,135,137,139–144,146,148,149,151,158,167,170]
Food security/Malnutrition	40	[4,11,14,31,35,38–40,43,44,46,48,52,60,75,98,99,101,102,111,113,114,116–121,123,124,126–128,132,139,142,147,166,168,172]
Loss of livelihood	8	[45,49,52,97,117,125,133,166]
Death	15	[10,50,57,58,60,62,63,71,72,80,82,84,88,92,163]
Other pathogens	26	[14,31,33,38,44,52,54,56,60,61,78,79,83,90,101,105,107,110,124,142–144,150,153,161]
Physical illnesses	32	[1,11,34,37,49,53–56,58,68,70,72–74,76,77,85,92,94,107,113,115,134,139,142,153,160,164,166,173]
Climate-induced displacement	6	[40,41,47,100,123,124]
Mental health (not limited to anxiety and depression)	13	[11,14,31,42,56,67,97,114,123,134,136,139,166]
Social determinant of health (including but not limited to social identity, attachment to place, safety, well-being)	7	[8,52,87,89,97,133,139]
Gender-based violence	2	[8,40]
HIV/AIDS	2	[68,131]

* Some studies covered multiple themes. Hence, N ≠ 153.

**Fig 2:**
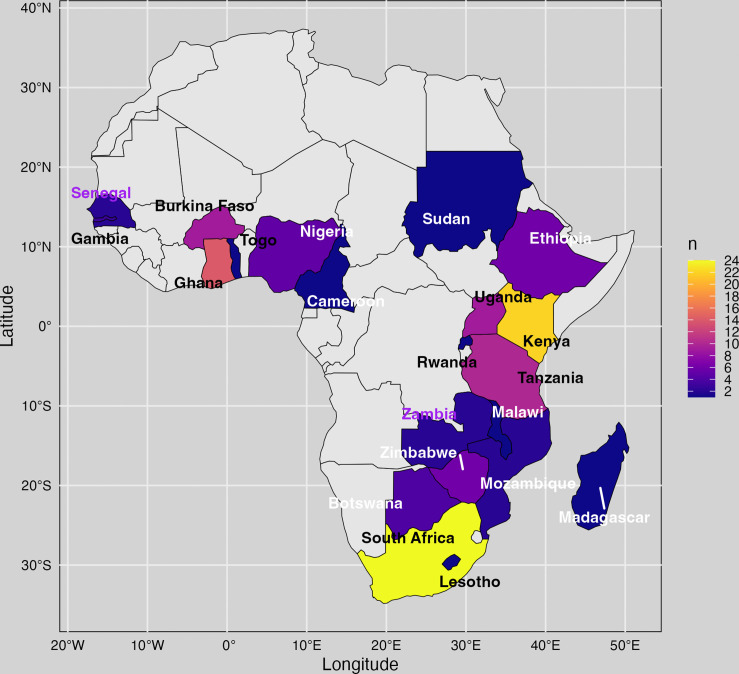
Map showing the number of climate change and health studies in sub-Saharan Africa, color-coded by research volume. Purple to yellow shades indicate more studies (2 to 24), while gray countries had no studies meeting the inclusion criteria (2001–2024). Only English-language publications were included.

This scoping review also identified specific countries in sub-Saharan Africa where climate change and health research has been conducted, aiming to highlight countries with insufficient research. Result shows that most of the studies were conducted in South and East Africa, with South Africa and Kenya having 24 and 22 published studies, respectively, followed by Ghana [14], Tanzania [10], and Burkina Faso [9]. Twenty-six studies included in this study were large scale studies conducted across multiple SSA countries (S1 Table). Only 24 out of 53 countries in sub-Saharan Africa have published studies on climate change and health over the past 24 years and 15 countries had less than 5 published studies on climate change and human health (Fig 2).

**Fig 3:**
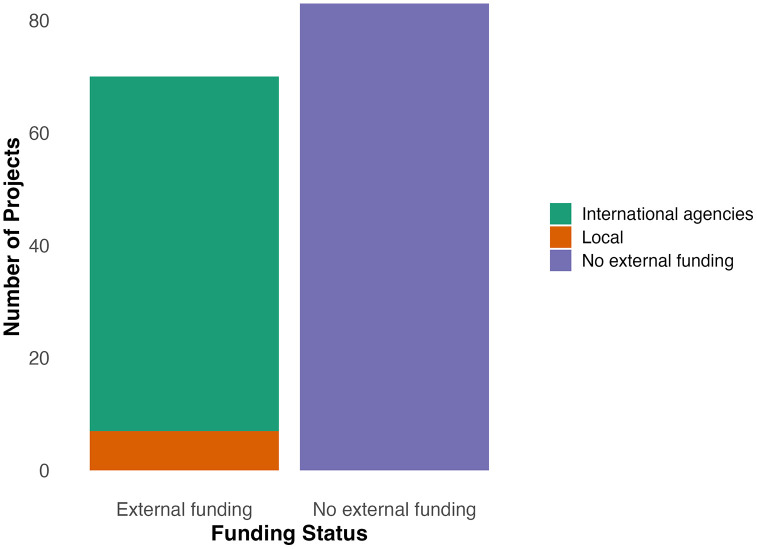
How are climate studies funded in sub-Saharan Africa.

Research funding sources were also a focus of this study. Of the studies considered, 45% (n = 69) were funded by external grants, while 55% (n = 84) were self-funded by researchers, declared as “no external funding” in research publication (Fig 3). Of the 45% (n = 69) of the research funded by external grants, 88.4% (n = 61) were grants provided by international agencies across North America, Europe and Asia, including but not limited to German Academic Exchange Services (DAAD), the Wellcome Trust, the Taiwan Ministry of Science and Technology Grant, the World Health Organization, the United States National Science Foundation, the Public Health Agency of Canada, the National Institutes of Health, the Rockefeller Foundation, the International Climate Change Information and Research Programme and Commonwealth. Funding was also provided by universities in the Global North, such as the University of Guelph. Only seven studies (11.6%) were funded through local funding provided by government agencies of sub-Saharan countries; four of those were provided by the South African Medical Research Council, one was a grant from Fundo Nacional de Investigação (FNI) in Mozambique, one was a grant from the Ethiopian Institute of Water Resources and one from the African Climate Change Fellowship Program (ACCFP).

Additionally, this study explored the impact of climate change on vulnerability and the health of vulnerable groups (S1 Fig). Vulnerable groups, such as children, the elderly, pregnant women, low-income populations, and those with pre-existing health conditions, are disproportionately affected by climate change due to factors like limited healthcare access, poor infrastructure, and heightened exposure to climate risks. Among the studies reviewed, 59 did not consider vulnerability in their research. Of those who did, 40 focused exclusively on children, while 23 addressed children along with other vulnerable groups, such as elderly individuals, pregnant women, disabled individuals, low-income groups, and immunocompromised individuals. Thirteen studies specifically examined gendered vulnerabilities, with 12 studies focused on women and 1 on men. Five studies exclusively focused on immunocompromised groups, 3 studies investigated the impacts of climate change on the elderly population, and an additional 2 investigated the effects on impoverished populations. Three studies focused on pregnant women, 1 study on farm workers, and 2 studies focused on mining workers. Finally, one study examined the impacts of climate change on the health of internally displaced populations and on local and Indigenous communities.

This research also explored the various adaptation strategies proposed to mitigate climate change impacts on SSA, as suggested by the authors of the included studies (S2 Fig). Thirty-seven studies (24%) had no proposed adaptation strategies. Adaptation strategies were grouped into themes including technology gaps, policy modifications, community-focused adaptation plans, climate preparedness programs and the need for funding. The majority of studies proposed a community-focused adaptation plan and the inclusion of local context in climate intervention (n = 37, 27%) as the most effective adaptation strategy to help residents of SSA minimize the impacts of climate change. Improved technology was the second highest proposed adaptation strategy suggested by the studies reviewed (n = 32, 23%), and the authors proposed technological improvements such as early warning systems, remote sensing, robust meteorological technologies, water treatment systems, vaccination, disease surveillance systems, energy-saving air conditioning, and climate-smart agricultural technologies. A total of 18% (n = 25) of the studies discussed inadequate local funding as a significant barrier to climate change adaptation and proposed an increased budget for climate-related concerns. Similarly, an additional 18% (n = 25) of the studies highlighted the need for climate preparedness programs among citizens, government officials and health workers. Finally, 14% (n = 20) of the studies further describe policy modification, specifically suggesting holistic and concerted efforts by individuals, multiple government agencies working together and donor agencies in the fight against climate change and resulting health impacts.

## Discussion

This study aimed to understand the relationship between climate change and health in SSA. Many of the events documented in the literature; such as seasonal droughts, floods, and heat episodes are part of long-standing and established weather patterns in the region, rather than direct consequences of anthropogenic climate change. However, these weather-health relationships are critically important for informing climate change adaptation strategies. As climate change is projected to increase the frequency, intensity, and unpredictability of extreme weather events, current health burdens linked to seasonal weather patterns can serve as proxies for anticipating future risks and vulnerabilities. The impact of climate change is already apparent in SSA, as the research reviewed in this study indicates that regions is already being affected by drought [60,113,118,125], extreme heat [9,53,57,59,63,166] and microbial and chemical water quality issues, leading to infectious diseases such as cholera and diarrhea outbreaks as a result of compounding climate extremes on water access and quality [9,16,56,135,140,146,174]. Additionally, there have been reports of natural disasters, such as floods [8,44,134,146] and cyclones [16], all contributing to negative health outcomes in addition to economic costs.

Research on climate change and health is critically important in SSA because the region faces heightened vulnerability due to a combination of environmental, social, and economic factors. Many countries in SSA are already experiencing the effects of climate variability that directly and indirectly impact human health [175]. At the same time, limited infrastructure, under-resourced health systems, high disease burdens, and widespread poverty constrain the capacity to respond to these challenges [176]. Understanding the intersection of climate and health in this context is essential for identifying risk pathways, informing adaptation strategies, and strengthening resilience.

The intensified climate events on the continent are leading to several physical, mental and emotional health impacts. Most of the studies in this review focused on WASH issues, food security, physical illnesses such as heat exhaustion, and pathogen infections such as cholera and malnutrition. However, the social, emotional and mental aspects of climate change have been relatively understudied. Loss of livelihood due to the impact of floods, extreme temperature and drought on agricultural, resource-based occupation and outdoor informal economies has been reported in a few studies in the sub-Saharan African context [52,133]. Loss of livelihood is often intertwined with disrupted community ties, loss of identity, climate-induced displacement, climate anxiety, depression and gender-based violence [40,41,52,123,166]. Therefore, climate change is leading to cascading outcomes affecting all aspects of health and wellness in the region.

There is an urgent need for accelerated climate change research in Africa, home to more than 1 billion people and experiencing rapid population growth. Despite the critical need for solutions, only 43 of the studies reviewed in this study discussed specific strategies and knowledge to mitigate the impact of climate change on health. These studies included contributions to new research, evaluations of existing mitigation efforts, and insights into issues unique to the continent, such as lack of access to basic services and nutrition. Several studies have provided specific mitigations, considerations, and recommendations. For example, a study in Ethiopia examined childhood diarrhea, finding a high incidence at the beginning of dry seasons [36]. Other studies have addressed climate-specific issues such as extreme temperatures, precipitation, and mortality and proposed mitigation steps to reduce hospital admissions, morbidity, and mortality [50,53,54], including within-hospital settings. The challenges related to WASH were also highlighted, with some studies identifying opportunities for integrating climate adaptation into WASH development planning [2]. The findings and recommendations from these studies could be implemented in other regions facing similar challenges. Research on water burdens and gender inequalities has emphasized specific strategies for reducing these disparities [115]. Overall, these studies offer valuable insights and recommendations that are specific to the region, that can be applied to mitigate the impact of climate change on health across SSA.

This review revealed key gaps and opportunities in climate change and health research in SSA. First, there are significant geographic disparities, with some countries being more frequently studied than others. This uneven distribution of research limits the ability to develop comprehensive, country-specific interventions and may lead to an incomplete understanding of regional needs. Additionally, while some studies adopt a multi-country or regional approach, aggregated data can obscure critical local disparities [177]. SSA is not a monolith; differences in climate zones, infrastructure, governance, and health system capacity create diverse risk profiles both across and within countries. Tailored, context-specific research is essential for designing effective adaptation strategies that reflect the unique vulnerabilities and strengths of individual communities [178]. Recognizing and addressing these intra-regional differences is key to promoting equitable and resilient responses to climate change. Furthermore, even in countries where research has been conducted, several authors emphasize the need for more primary climate and health data to support evidence-based decision-making [14,128,171]. Expanding research efforts to include understudied countries can provide a more comprehensive understanding of climate change and health impact across SSA, ensuring that attention is given to those most impacted by climate change and the opportunity to learn from each other.

There are key considerations that need to be taken into account when discussing climate change solutions for SSA. For instance, we need to integrate and discuss social determinants of health as we think of climate change and health. Only five studies highlighted or discussed the associations between health, social determinants of health, and climate change [8,52,97,133,139]. However, it is well understood that the work a person does, where people live, their educational level, and income level all play a role in determining health [179]. More research is needed to account for climate change as a determinant of health, especially since low-income countries will be impacted the most. Additionally, there are only a few studies on how climate change leads to gender-based violence. However, relationships between climate change, loss of livelihood, feelings of emasculation and gender-based violence have been reported in sub-Saharan Africa [8,40].

Many studies have not adequately considered the vulnerability of different population groups through an intersectional lens. While several papers acknowledged groups such as children, pregnant women, and the elderly as vulnerable, these mentions were often superficial, and 38 studies did not consider vulnerability at all. This gap highlights a pressing need for more comprehensive research that accounts for the diverse experiences and needs within communities. Climate change does not impact all people equally and its effects are shaped by existing social, economic, and environmental inequalities [180]. Understanding community heterogeneity and the overlapping vulnerabilities faced by marginalized groups is essential for developing effective adaptation and mitigation strategies. Policies must be revised to move beyond a one-size-fits-all approach and instead prioritize equity by identifying and addressing the specific needs of those most at risk. This includes integrating data on gender, age, income level, disability, and social exclusion into climate-health planning [180]. Incorporating vulnerability ensures that resources are allocated more fairly, that interventions are more effective, and that the resilience of entire communities is strengthened and not just the most visible or privileged segments. Ultimately, placing vulnerability at the center of climate-health policy is not only a matter of justice but also a practical necessity for sustainable and inclusive development in SSA.

Further, while some climate themes have been extensively studied, others remain underexplored and require greater attention. There is a notable lack of research in areas such as extreme heat, natural disasters, and air pollution. For instance, only 10 studies highlighted research related to air pollution [31–33,96,113,162,163], and just two specifically addressed the intersection of climate change and cardiovascular diseases [96,115]. This is a significant gap, especially considering that the Lancet Global Burden of Disease (2016) reported that 33% of the global burden of stroke is attributed to environmental factors such as air pollution and lead exposure [181]. Air pollution contributes to climate change through the release of greenhouse gases and short-lived climate pollutants, but it can also be worsened by climate-related phenomena such as wildfires or stagnant air masses during heatwaves. This study also revealed that air pollution has emerged as a significant contributor to the global burden of stroke in low- and middle-income countries (LMIC) [181]. Climate change is expected to increase drought and air pollution in some regions of the world, including SSA [182]. Despite this significance, research in this area is almost nonexistent and requires urgent attention.

A few other studies offer concrete pathways and recommendations on what countries need to focus on and prioritize to reduce the impact of climate change. Technological advancement was one of the most common emerging themes from the included studies, although implementing the suggested technologies might be far-fetched in SSA landscapes. For example, in areas where infectious diseases are a concern, authors recommend focusing on early warning signs, disease forecasting, and climate disaster mitigation [14,128] to strengthen public health systems to cope with and reduce climate change impacts. However, it is difficult in low-income countries to focus on early warning systems or disease forecasting when basic solutions for preventing diseases, such as access to safe and clean water or sanitation, are still lacking.

For instance, without the additional burden of intensified climate impacts, current water and sanitation infrastructure or healthcare infrastructure is inadequate to protect public health [2,17,19,104,140]. Some countries are already experiencing preventable waterborne diseases that could be addressed by having adequate water and sanitation infrastructure. Some examples include the cholera outbreak in Tanzania in 2023, ongoing in 2024, and another in June 2024 in Nigeria [16,18,178–180], both attributed to climate change. In addition, lack of technologies and inadequate electricity coverage create barriers to implementing other simple solutions to combat extreme heat conditions [166] and a lack of improved agricultural technologies to combat food insecurity [4]. For example, irrigation systems are creating vulnerabilities further intensified by climate change. The continent must catch up to address current poor living conditions and, at the same time, accelerate the research needed to move to the 21^st^ century to address climate change issues.

In tandem with inadequate climate policies, a lack of funding was also identified as limiting climate change research progress in SSA. In the present study, more than half of the studies reviewed were self-funded by researchers. The large amount of self-funded research highlights the financial constraints researchers face, which can limit the scope, scale, and depth of their studies, ultimately hindering the development of comprehensive climate change mitigation and adaptation strategies. Additionally, only seven of the 153 selected studies were funded by local governmental agencies, revealing a much deeper systemic issue about the perceived relevance of climate change impacts in this region. Therefore, there is an urgent need for increased investments in climate research [2,19,20,52,101,114,127]. Funding climate research will help inform climate decision-making and policies [128]. There is also a lack of climate consideration in government policies [2]. To ensure that climate investment and increased funding reap the maximum benefits, there is a need for multiagency collaboration between government agencies to inform policies that achieve economic, environmental, social and health justice [97,173]. Local residents and external donor agencies should also be included in climate strategizing to ensure maximum benefits [68]. Addressing these limitations through enhanced funding, comprehensive policies, and inclusive collaboration will be pivotal for effectively mitigating the impacts of climate change in SSA.

Even with limited resources, countries can strategize and prioritize actions towards climate change. One thing is evident: the issue is not a lack of understanding of the urgency to address climate change. Studies evaluating climate change perception in various African countries have shown that most of the population is familiar with climate change and have experienced it to some extent [14,41,100,125,127,183]. However, there is insufficient data due to technological and resource limitations to provide tailored interventions at the community and national, and lack of national policies. For instance, in Zimbabwe, following the devastation caused by Cyclone Ida, natural disasters exposed another problem—food insecurity. A study of 19 health facilities conducted after Cyclone Ida revealed that 94% of the facilities were not equipped to address malnutrition, either due to lack of proper training, inadequate staffing, or failure to follow proper protocols [126]. With climate change, areas with limited resources will be most affected, and there is a need for adequate systems to address climate change and health issues. Therefore, community-focused adaptation plans are necessary, taking into account the strengths and vulnerabilities of individual communities and countries. These details can only be revealed through community-level or national research, which is often obscured by large-resolution, aggregated datasets produced by major development agencies.

There is also a dire need to build capacity for health sector personnel through climate preparedness programs that integrate climate resilience into public health systems. Educational and research institutions can offer expertise in addressing regional or national climate change solutions. These programs should focus on enhancing the ability of healthcare infrastructure to withstand climate-related events and training healthcare professionals to respond effectively to climate-induced health issues [6,38,100,107,147,182,184]. Additionally, community-based interventions are essential for educating and empowering local populations on climate-related health risks and adaptive practices. Incorporating climate education in formal and informal places of learning in communities, has shown to increase ability to identify vulnerability and build adaptive capacity [38,42]. These climate education programs may include utilizing traditional knowledge to inform strategies to reduce individual- and community-level vulnerabilities, improve infrastructure, create multiple climate-resilient income streams, and address the intersectional vulnerabilities that climate change exposes [42]. Due to the large population of young people in Africa, strategies targeting young people are paramount [41]. Educational programs may also target improved monitoring and surveillance systems [14,171] to fill the data gap and improve data-driven decision-making. These efforts will ensure that public health systems are resilient, adaptive, and equipped to protect the well-being of all individuals, especially the most vulnerable populations.

Although this paper has several merits, including the identification of key interconnections between climate and health in sub-Saharan Africa, it is not without limitations. One major limitation is the inclusion criterion of English-language publications only. This introduces a language bias that may underrepresent research conducted in Francophone, Lusophone (Portuguese-speaking), and Arabic-speaking countries. As a result, these regions may be incorrectly characterized as lacking in climate and health research, when in fact relevant studies may exist but were excluded due to language barriers. Additionally, this review excluded studies focused solely on malaria due to the extensive and well-established body of literature on the subject. While this decision allowed for the exploration of underexamined health outcomes, it may also omit key climate-sensitive disease trends that remain highly relevant to public health in the region.

## Conclusion and recommendations

This scoping review reveals a substantial and growing body of literature addressing the intersection of climate change and health in SSA, but it also highlights significant gaps in research, policy implementation, and resource allocation. While there has been progress in understanding climate-related health outcomes—particularly regarding WASH, food security, and infectious diseases—other critical issues, such as mental health, gender-based violence, and the health impacts of air pollution and extreme heat, remain severely underexplored. The uneven geographic distribution of studies, predominance of self-funded research, lack of localized data, and limited attention to vulnerable populations reflect systemic constraints that impede effective climate adaptation and public health response in SSA. Despite widespread awareness of climate change impacts among local populations, there remains a critical need for actionable data, tailored adaptation strategies, and sustainable institutional support to protect public health in the face of a changing climate

To address these challenges, we recommend the following: 1) Expand geographic and context-specific research by prioritizing funding in understudied countries and tailor research to local vulnerabilities; 2) Invest in capacity building by integrating climate training into public health and medical education and establish preparedness programs for health professionals; 3) Increase local and national funding by encouraging national governments to fund climate-health research and reduce reliance on external donors; 4) Mainstream vulnerability and equity by adopting intersectional approaches in policy and research, to address the needs of marginalized groups; 5) Strengthen data and surveillance systems to enhance real-time data collection and support evidence-based policy-making tailored to local contexts; 6) Integrate climate considerations into development planning by embedding them into national health, agriculture, and disaster response agendas; and 7) Support community-led adaptation by leveraging traditional knowledge locally driven sustainable strategies.

Implementing these recommendations will support SSA in developing inclusive, equitable, and resilient climate-health strategies essential for long-term public health protection.

## Supporting information

S1 Fig Vulnerable Groups Identified in Past Studies and the Number of times the group was mentioned. (TIF)

S2 Fig Adaptation Strategies Suggested by Authors in Current Study to Address Climate Change and Health Issues in Sub-Saharan Africa. (TIF)

S1 Table Number of Studies on Climate Change and Health by Country in Sub-Saharan Africa. (DOCX)

S1 Checklist PRISMA 2020 checklist October 2025. (DOCX)

## References

[1] Alexander KA, Carzolio M, Goodin D, Vance E. Climate change is likely to worsen the public health threat of diarrheal disease in Botswana. Int J Environ Res Public Health. 2013;10(4):1202–30. doi: 10.3390/ijerph10041202 23531489 PMC3709313

[2] Alhassan S, Hadwen WL. Challenges and Opportunities for Mainstreaming Climate Change Adaptation into WaSH Development Planning in Ghana. Int J Environ Res Public Health. 2017;14(7):749. doi: 10.3390/ijerph14070749 28698518 PMC5551187

[3] Brauer M, Roth GA, Aravkin AY, Zheng P, Abate KH, Abate YH, et al. Global burden and strength of evidence for 88 risk factors in 204 countries and 811 subnational locations, 1990–2021: a systematic analysis for the Global Burden of Disease Study 2021. The Lancet [Internet]. 2024 May [cited 2024 Jun 19];403(10440):2162–203. Available from: https://linkinghub.elsevier.com/retrieve/pii/S0140673624009334 10.1016/S0140-6736(24)00933-4 PMC11120204 38762324

[4] Mugambiwa SS, Tirivangasi HM. Climate change: A threat towards achieving “Sustainable Development Goal number two” (end hunger, achieve food security and improved nutrition and promote sustainable agriculture) in South Africa. Jamba. 2017;9(1):350. doi: 10.4102/jamba.v9i1.350 29955332 PMC6014178

[5] Myers J, Young T, Galloway M, Manyike P, Tucker T. A public health approach to the impact of climate change on health in southern Africa - identifying priority modifiable risks. S Afr Med J. 2011;101(11):817–20. 22272963

[6] Negev M, Teschner N, Rosenthal A, Levine H, Lew-Levy C, Davidovitch N. Adaptation of health systems to climate-related migration in Sub-Saharan Africa: Closing the gap. International Journal of Hygiene and Environmental Health [Internet]. 2019 Mar [cited 2023 Oct 15];222(2):311–4. Available from: https://linkinghub.elsevier.com/retrieve/pii/S1438463918304991 30503929 10.1016/j.ijheh.2018.10.004

[7] World Meteorological Organization. World Meteorological Organization. 2023 [cited 2024 Jun 19]. Africa suffers disproportionately from climate change. Available from: https://wmo.int/media/news/africa-suffers-disproportionately-from-climate-change

[8] Allen EM, Munala L, Henderson JR. Kenyan Women Bearing the Cost of Climate Change. Int J Environ Res Public Health. 2021;18(23):12697. doi: 10.3390/ijerph182312697 34886422 PMC8656926

[9] Chersich MF, Wright CY, Venter F, Rees H, Scorgie F, Erasmus B. Impacts of Climate Change on Health and Wellbeing in South Africa. Int J Environ Res Public Health. 2018;15(9):1884. doi: 10.3390/ijerph15091884 30200277 PMC6164733

[10] Wichmann J. Heat effects of ambient apparent temperature on all-cause mortality in Cape Town, Durban and Johannesburg, South Africa: 2006-2010. Sci Total Environ. 2017;587–588:266–72. 10.1016/j.scitotenv.2017.02.135 28242220

[11] Zhu Y, He C, Gasparrini A, Vicedo-Cabrera AM, Liu C, Bachwenkizi J, et al. Global warming may significantly increase childhood anemia burden in sub-Saharan Africa. One Earth. 2023;6(10):1388–99. doi: 10.1016/j.oneear.2023.09.003 37904727 PMC7615260

[12] Selassie AA, Fuje H. IMF. 2021 [cited 2024 Jun 19]. Seven Charts that Show Sub-Saharan Africa at a Crucial Point. Available from: https://www.imf.org/en/News/Articles/2021/10/20/na102021-seven-charts-that-show-sub-saharan-africa-at-a-crucial-point

[13] Baptista DMS, Farid M, Fayad D, Kemoe L, Lanci LS, Mitra P. IMF. 2022 [cited 2024 Jun 19]. Climate Change and Chronic Food Insecurity in Sub-Saharan Africa in: Departmental Papers Volume 2022 Issue 016 (2022). Available from: https://www.elibrary.imf.org/view/journals/087/2022/016/article-A001-en.xml

[14] Opoku SK, Leal Filho W, Hubert F, Adejumo O. Climate Change and Health Preparedness in Africa: Analysing Trends in Six African Countries. Int J Environ Res Public Health. 2021;18(9):4672. doi: 10.3390/ijerph18094672 33925753 PMC8124714

[15] United Nations. Africa Renewal. 2021 [cited 2024 Jun 19]. Climate change triggers food insecurity, poverty and displacement in Africa. Available from: https://www.un.org/africarenewal/magazine/climate-change-triggers-food-insecurity-poverty-and-displacement-africa

[16] Cambaza E, Mongo E, Anapakala E, Nhambire R, Singo J, Machava E. Outbreak of cholera due to cyclone Kenneth in northern Mozambique, 2019. Int J Environ Res Public Health. 2019;16(16). 10.3390/ijerph16162925 PMC6720437 31443180

[17] Ebi KL, Hess JJ, Watkiss P. Health Risks and Costs of Climate Variability and Change. 2017. 30212118

[18] Mendelsohn J, Dawson T. Climate and cholera in KwaZulu-Natal, South Africa: the role of environmental factors and implications for epidemic preparedness. Int J Hyg Environ Health. 2008;211(1–2):156–62. doi: 10.1016/j.ijheh.2006.12.002 17383231

[19] Olago D, Marshall M, Wandiga SO, Opondo M, Yanda PZ, Kanalawe R, et al. Climatic, socio-economic, and health factors affecting human vulnerability to cholera in the Lake Victoria basin, East Africa. Ambio. 2007;36(4):350–8. doi: 10.1579/0044-7447(2007)36[350:csahfa]2.0.co;2 17626474

[20] Trærup SLM, Ortiz RA, Markandya A. The Costs of Climate Change: A Study of Cholera in Tanzania. IJERPH [Internet]. 2011 Nov 28 [cited 2023 Dec 22];8(12):4386–405. Available from: http://www.mdpi.com/1660-4601/8/12/4386 10.3390/ijerph8124386 PMC3290983 22408580

[21] Coates SJ, Enbiale W, Davis MDP, Andersen LK. The effects of climate change on human health in Africa, a dermatologic perspective: a report from the International Society of Dermatology Climate Change Committee. Int J Dermatol. 2020;59(3):265–78. doi: 10.1111/ijd.14759 31970754

[22] Hussey LK, Arku G. Are we ready for it? Health systems preparedness and capacity towards climate change-induced health risks: perspectives of health professionals in Ghana. Climate and Development. 2019;12(2):170–82. doi: 10.1080/17565529.2019.1610350

[23] Ruhweza A. Africa Renewal. 2022 [cited 2024 Jun 19]. Climate adaptation: Castle in the sky or achievable reality? Available from: https://www.un.org/africarenewal/magazine/november-2022/climate-adaptation-castle-sky-or-achievable-reality

[24] Byass P. Climate change and population health in Africa: where are the scientists? Global Health Action [Internet]. 2009 Nov 11 [cited 2024 Jun 19];2(1):2065. Available from: 10.3402/gha.v2i0.2065 PMC2799228 20052421

[25] Odey EA, Abo BO, Li Z, Zhou X, Giwa AS. Influence of climate and environmental change in Nigeria: a review on vulnerability and adaptation to climate change. Reviews on Environmental Health [Internet]. 2018 Dec 1 [cited 2024 Jun 19];33(4):441–7. Available from: https://www.degruyter.com/document/doi/10.1515/reveh-2018-0043/html 10.1515/reveh-2018-0043 30291786

[26] Azour J, Selassie AA. IMF. 2023 [cited 2024 Jun 19]. Africa’s Fragile States Are Greatest Climate Change Casualties. Available from: https://www.imf.org/en/Blogs/Articles/2023/08/30/africas-fragile-states-are-greatest-climate-change-casualties

[27] African Development Bank. African Development Bank Group. African Development Bank Group; 2019 [cited 2024 Jun 19]. Climate Change in Africa. Available from: https://www.afdb.org/en/cop25/climate-change-africa

[28] U. N. Environment. UNEP - UN Environment Programme. 2017 [cited 2024 Jun 19]. Responding to climate change. Available from: http://www.unep.org/regions/africa/regional-initiatives/responding-climate-change

[29] Covidence systematic review [Internet]. Melborne, Australia; Available from: www.covidence.org

[30] Watson RT, Albritton DL, Intergovernmental Panel on Climate Change, Intergovernmental Panel on Climate Change, Intergovernmental Panel on Climate Change, editors. Climate change 2001: synthesis report. Cambridge; New York: Cambridge University Press; 2001. 397 p.

[31] Dos Santos M, John J, Garland R, Palakatsela R, Banos A, Martens P, et al. Climate change and health within the South African context: A thematic content analysis study of climate change and health expert interviews. African Journal of Primary Health Care & Family Medicine [Internet]. 2022 Mar 30 [cited 2023 Oct 15];14(1). Available from: http://www.phcfm.org/index.php/PHCFM/article/view/3023 10.4102/phcfm.v14i1.3203 PMC8991177 35384686

[32] Pedder H, Kapwata T, Howard G, Naidoo RN, Kunene Z, Morris RW, et al. Lagged Association between Climate Variables and Hospital Admissions for Pneumonia in South Africa. Int J Environ Res Public Health. 2021;18(12):6191. doi: 10.3390/ijerph18126191 34201085 PMC8228646

[33] Thompson AA, Matamale L, Kharidza SD. Impact of Climate Change on Children’s Health in Limpopo Province, South Africa. IJERPH [Internet]. 2012 Mar 8 [cited 2023 Oct 15];9(3):831–54. Available from: http://www.mdpi.com/1660-4601/9/3/831 10.3390/ijerph9030831 PMC3367281 22690167

[34] Odunola O, Odunsi O, Daramola O. Climate change evidence and effects of climate-change-related diseases on children’s health. Environ Qual Manage [Internet]. 2018;28(1):47–55. Available from: https://www.scopus.com/inward/record.uri?eid=2-s2.0-85053780899&doi=10.1002%2ftqem.21571&partnerID=40&md5=2d1388055eae73341ebd4435336c8399

[35] Abayomi A, Cowan MN. The HIV/AIDS epidemic in South Africa: Convergence with tuberculosis, socioecological vulnerability, and climate change patterns. South African Medical Journal [Internet]. 2014;104(8):583. Available from: https://www.embase.com/search/results?subaction=viewrecord&id=L373677268&from=export 26307805 10.7196/samj.8645

[36] Azage M, Kumie A, Worku A, C. Bagtzoglou A, Anagnostou E. Effect of climatic variability on childhood diarrhea and its high risk periods in northwestern parts of Ethiopia. Shaman J, editor. PLoS ONE [Internet]. 2017 Oct 26 [cited 2023 Dec 22];12(10):e0186933. Available from: https://dx.plos.org/10.1371/journal.pone.0186933 10.1371/journal.pone.0186933 PMC5658103 29073259

[37] Ephraim RKD, Asamoah CA, Abaka-Yawson A, Kwadzokpui PK, Adusei S. Climate change causes changes in biochemical markers of kidney disease. BMC Nephrol. 2020;21(1):542. doi: 10.1186/s12882-020-02186-w 33308177 PMC7733275

[38] Kowalcyk M, Dorevitch S. A Framework for Evaluating Local Adaptive Capacity to Health Impacts of Climate Change: Use of Kenya’s County-Level Integrated Development Plans. Ann Glob Health. 2024;90(1):15. doi: 10.5334/aogh.4266 38370864 PMC10870949

[39] Lloyd SJ, Kovats RS, Chalabi Z. Climate change, crop yields, and undernutrition: development of a model to quantify the impact of climate scenarios on child undernutrition. Environ Health Perspect. 2011;119(12):1817–23. doi: 10.1289/ehp.1003311 21844000 PMC3261974

[40] Munala L, Allen EM, Frederick AJ, Ngũnjiri A. Climate Change, Extreme Weather, and Intimate Partner Violence in East African Agrarian-Based Economies. Int J Environ Res Public Health. 2023;20(23). 10.3390/ijerph20237124 PMC10706456 38063554

[41] Odonkor ST, Dei EN, Sallar AM. Knowledge, Attitude, and Adaptation to Climate Change in Ghana. ScientificWorldJournal. 2020;2020:3167317. doi: 10.1155/2020/3167317 33299383 PMC7710404

[42] Prencipe L, Houweling TAJ, van Lenthe FJ, Kajula L, Palermo T. Climate distress, climate-sensitive risk factors, and mental health among Tanzanian youth: a cross-sectional study. Lancet Planet Health. 2023;7(11):e877–87. doi: 10.1016/S2542-5196(23)00234-6 37940208

[43] Rakotoarison N, Raholijao N, Razafindramavo LM, Rakotomavo ZAPH, Rakotoarisoa A, Guillemot JS, et al. Assessment of Risk, Vulnerability and Adaptation to Climate Change by the Health Sector in Madagascar. Int J Environ Res Public Health. 2018;15(12):2643. doi: 10.3390/ijerph15122643 30486244 PMC6313613

[44] Sorgho R, Jungmann M, Souares A, Danquah I, Sauerborn R. Climate Change, Health Risks, and Vulnerabilities in Burkina Faso: A Qualitative Study on the Perceptions of National Policymakers. IJERPH [Internet]. 2021 May 7 [cited 2023 Oct 15];18(9):4972. Available from: https://www.mdpi.com/1660-4601/18/9/4972 10.3390/ijerph18094972 PMC8125418 34067050

[45] Sverdlik A, Kothiwal K, Kadungure A, Agarwal S, Machemedze R, Verma S, et al. Understanding the interplay of occupational, public health, and climate-related risks for informal workers: A new framework with findings from Zimbabwe and India. Soc Sci Med. 2024;348:116750. doi: 10.1016/j.socscimed.2024.116750 38531215

[46] van Bavel B, Ford LB, King R, Lwasa S, Namanya D, Twesigomwe S, et al. Integrating climate in Ugandan health and subsistence food systems: where diverse knowledges meet. BMC Public Health. 2020;20(1):1864. doi: 10.1186/s12889-020-09914-9 33276748 PMC7718713

[47] Kelly-Hope LA, Harding-Esch EM, Willems J, Ahmed F, Sanders AM. Conflict-climate-displacement: A cross-sectional ecological study determining the burden, risk and need for strategies for neglected tropical disease programmes in Africa. BMJ Open [Internet]. 2023;13(5). Available from: https://www.embase.com/search/results?subaction=viewrecord&id=L2024678132&from=export 10.1136/bmjopen-2023-071557 PMC10193097 37197807

[48] van der Merwe E, Clance M, Yitbarek E. Climate change and child malnutrition: A Nigerian perspective. Food Policy [Internet]. 2022;113. Available from: https://www.scopus.com/inward/record.uri?eid=2-s2.0-85131834080&doi=10.1016%2fj.foodpol.2022.102281&partnerID=40&md5=fcadda2642fd86beedb0eeca69fe2b4b

[49] Nsengiyumva R, Mukarubayiza MR, Murekatete C, Meharry P. Climate Change Associated with Neonatal Health Risks: Rwandan Nurses and Midwives’ Awareness and Perceptions. Rwanda J Med Health Sci [Internet]. 2020;3(2):261–72. Available from: https://www.scopus.com/inward/record.uri?eid=2-s2.0-85151013660&doi=10.4314%2frjmhs.v3i2.15&partnerID=40&md5=651f55b581f4cda035ffc0f7608cb0c4

[50] Arisco NJ, Sewe MO, Bärnighausen T, Sié A, Zabre P, Bunker A. The effect of extreme temperature and precipitation on cause-specific deaths in rural Burkina Faso: a longitudinal study. Lancet Planet Health. 2023;7(6):e478–89. doi: 10.1016/S2542-5196(23)00027-X 37286245

[51] Barteit S, Boudo V, Ouedraogo A, Zabré P, Ouremi L, Sié A, et al. Feasibility, acceptability and validation of wearable devices for climate change and health research in the low-resource contexts of Burkina Faso and Kenya: Study protocol. PLoS One. 2021;16(9):e0257170. doi: 10.1371/journal.pone.0257170 34591893 PMC8483291

[52] Berrang-Ford L, Dingle K, Ford JD, Lee C, Lwasa S, Namanya DB, et al. Vulnerability of indigenous health to climate change: a case study of Uganda’s Batwa Pygmies. Soc Sci Med. 2012;75(6):1067–77. doi: 10.1016/j.socscimed.2012.04.016 22703884

[53] Bidassey-Manilal S, Wright CY, Kapwata T, Shirinde J. A Study Protocol to Determine Heat-Related Health Impacts among Primary Schoolchildren in South Africa. Int J Environ Res Public Health. 2020;17(15):5531. doi: 10.3390/ijerph17155531 32751802 PMC7432321

[54] Bishop-Williams KE, Berrang-Ford L, Sargeant JM, Pearl DL, Lwasa S, Namanya DB, et al. Understanding Weather and Hospital Admissions Patterns to Inform Climate Change Adaptation Strategies in the Healthcare Sector in Uganda. Int J Environ Res Public Health. 2018;15(11):2402. doi: 10.3390/ijerph15112402 30380686 PMC6265697

[55] Bonell A, Sonko B, Badjie J, Samateh T, Saidy T, Sosseh F, et al. Environmental heat stress on maternal physiology and fetal blood flow in pregnant subsistence farmers in The Gambia, west Africa: an observational cohort study. Lancet Planet Health. 2022;6(12):e968–76. doi: 10.1016/S2542-5196(22)00242-X 36495891 PMC9756110

[56] Codjoe SNA, Gough KV, Wilby RL, Kasei R, Yankson PWK, Amankwaa EF, et al. Impact of extreme weather conditions on healthcare provision in urban Ghana. Soc Sci Med. 2020;258:113072. doi: 10.1016/j.socscimed.2020.113072 32502835

[57] Diboulo E, Sié A, Rocklöv J, Niamba L, Yé M, Bagagnan C, et al. Weather and mortality: a 10 year retrospective analysis of the Nouna Health and Demographic Surveillance System, Burkina Faso. Glob Health Action. 2012;5:6–13. doi: 10.3402/gha.v5i0.19078 23195510 PMC3508665

[58] Fotso-Nguemo TC, Vondou DA, Diallo I, Diedhiou A, Weber T, Tanessong RS, et al. Potential impact of 1.5, 2 and 3 °C global warming levels on heat and discomfort indices changes over Central Africa. Sci Total Environ. 2022;804:150099. 10.1016/j.scitotenv.2021.150099 34517321

[59] Frimpong K, Van Etten E J E, Oosthuzien J, Fannam Nunfam V, MPHIL Development Studies. Heat exposure on farmers in northeast Ghana. Int J Biometeorol. 2017;61(3):397–406. doi: 10.1007/s00484-016-1219-7 27498220

[60] Hassan M, Saif K, Ijaz MS, Sarfraz Z, Sarfraz A, Robles-Velasco K, et al. Mean Temperature and Drought Projections in Central Africa: A Population-Based Study of Food Insecurity, Childhood Malnutrition and Mortality, and Infectious Disease. Int J Environ Res Public Health. 2023;20(3):2697. doi: 10.3390/ijerph20032697 36768062 PMC9915533

[61] Hussey LK, Arku G. Conceptualizations of climate-related health risks among health experts and the public in Ghana. Soc Sci Med. 2019;223:40–50. doi: 10.1016/j.socscimed.2019.01.026 30708170

[62] Kapwata T, Gebreslasie MT, Mathee A, Wright CY. Current and Potential Future Seasonal Trends of Indoor Dwelling Temperature and Likely Health Risks in Rural Southern Africa. Int J Environ Res Public Health. 2018;15(5):952. doi: 10.3390/ijerph15050952 29755105 PMC5981991

[63] Kapwata T, Abdelatif N, Scovronick N, Gebreslasie MT, Acquaotta F, Wright CY. Identifying heat thresholds for South Africa towards the development of a heat-health warning system. Int J Biometeorol. 2024;68(2):381–92. doi: 10.1007/s00484-023-02596-z 38157021 PMC10794383

[64] Koch M, Matzke I, Huhn S, Sié A, Boudo V, Compaoré G, et al. Assessing the Effect of Extreme Weather on Population Health Using Consumer-Grade Wearables in Rural Burkina Faso: Observational Panel Study. JMIR Mhealth Uhealth. 2023;11:e46980. doi: 10.2196/46980 37938879 PMC10666008

[65] Longbottom J, Caminade C, Gibson HS, Weiss DJ, Torr S, Lord JS. Modelling the impact of climate change on the distribution and abundance of tsetse in Northern Zimbabwe. Parasit Vectors. 2020;13(1):526. doi: 10.1186/s13071-020-04398-3 33076987 PMC7574501

[66] Lusambili A, Khaemba P, Agoi F, Oguna M, Nakstad B, Scorgie F, et al. Process and outputs from a community codesign workshop on reducing impact of heat exposure on pregnant and postpartum women and newborns in Kilifi, Kenya. Front Public Health. 2023;11:1146048. doi: 10.3389/fpubh.2023.1146048 37719738 PMC10501312

[67] Ndetei DM, Wasserman D, Mutiso V, Shanley JR, Musyimi C, Nyamai P, et al. The perceived impact of climate change on mental health and suicidality in Kenyan high school students. BMC Psychiatry. 2024;24(1):117. doi: 10.1186/s12888-024-05568-8 38347450 PMC10860278

[68] Nkanga MSN, Longo-Mbenza B, Adeniyi OV, Ngwidiwo JB, Katawandja AL, Kazadi PRB, et al. Ageing, exposure to pollution, and interactions between climate change and local seasons as oxidant conditions predicting incident hematologic malignancy at KINSHASA University clinics, Democratic Republic of CONGO (DRC). BMC Cancer. 2017;17(1):559. doi: 10.1186/s12885-017-3547-3 28835214 PMC5569529

[69] Nnko HJ, Gwakisa PS, Ngonyoka A, Sindato C, Estes AB. Potential impacts of climate change on geographical distribution of three primary vectors of African Trypanosomiasis in Tanzania’s Maasai Steppe: G. m. morsitans, G. pallidipes and G. swynnertoni. PLoS Negl Trop Dis. 2021;15(2):e0009081. doi: 10.1371/journal.pntd.0009081 33571190 PMC7904224

[70] Nunfam VF, Van Etten EJ, Oosthuizen J, Adusei-Asante K, Frimpong K. Climate change and occupational heat stress risks and adaptation strategies of mining workers: Perspectives of supervisors and other stakeholders in Ghana. Environ Res. 2019;169:147–55. doi: 10.1016/j.envres.2018.11.004 30458350

[71] Nyadanu SD, Tessema GA, Mullins B, Kumi-Boateng B, Ofosu AA, Pereira G. Prenatal exposure to long-term heat stress and stillbirth in Ghana: A within-space time-series analysis. Environ Res. 2023;222:115385. doi: 10.1016/j.envres.2023.115385 36736550

[72] Pasquini L, Van Aardenne L, Godsmark CN, Lee J, Jack C. Emerging climate change-related public health challenges in Africa: A case study of the heat-health vulnerability of informal settlement residents in Dar es Salaam, Tanzania. Science of The Total Environment [Internet]. 2020 Dec [cited 2023 Oct 15];747:141355. Available from: https://linkinghub.elsevier.com/retrieve/pii/S0048969720348841 10.1016/j.scitotenv.2020.141355 32777515

[73] Scorgie F, Lusambili A, Luchters S, Khaemba P, Filippi V, Nakstad B, et al. “Mothers get really exhausted!” The lived experience of pregnancy in extreme heat: Qualitative findings from Kilifi, Kenya. Soc Sci Med. 2023;335:116223. doi: 10.1016/j.socscimed.2023.116223 37725839

[74] Spencer S, Samateh T, Wabnitz K, Mayhew S, Allen H, Bonell A. The Challenges of Working in the Heat Whilst Pregnant: Insights From Gambian Women Farmers in the Face of Climate Change. Front Public Health. 2022;10:785254. doi: 10.3389/fpubh.2022.785254 35237548 PMC8883819

[75] Tusting LS, Bradley J, Bhatt S, Gibson HS, Weiss DJ, Shenton FC, et al. Environmental temperature and growth faltering in African children: a cross-sectional study. Lancet Planet Health. 2020;4(3):e116–23. doi: 10.1016/S2542-5196(20)30037-1 32220673 PMC7232952

[76] Wright CY, Dominick F, Kapwata T, Bidassey-Manilal S, Engelbrecht JC, Stich H, et al. Socio-economic, infrastructural and health-related risk factors associated with adverse heat-health effects reportedly experienced during hot weather in South Africa. Pan Afr Med J. 2019;34:40. doi: 10.11604/pamj.2019.34.40.17569 31762907 PMC6859010

[77] Matzke I, Huhn S, Koch M, Maggioni MA, Munga S, Muma JO, et al. Assessment of Heat Exposure and Health Outcomes in Rural Populations of Western Kenya by Using Wearable Devices: Observational Case Study. JMIR mHealth and uHealth [Internet]. 2024;12((Matzke I.; Huhn S.; Koch M.; Bärnighausen T.; Dambach P.; Barteit S.) Heidelberg Institute of Global Health, Heidelberg University Hospital, Heidelberg University, Heidelberg, Germany(Maggioni M.A.) Charité-Universitätsmedizin Berlin, Institute of Phys):e54669. Available from: https://www.embase.com/search/results?subaction=viewrecord&id=L644684763&from=export 10.2196/54669 PMC11258525 38963698

[78] Situma S, Nyakarahuka L, Omondi E, Mureithi M, Mweu MM, Muturi M, et al. Widening geographic range of Rift Valley fever disease clusters associated with climate change in East Africa. BMJ Global Health [Internet]. 2024;9(6). Available from: https://www.embase.com/search/results?subaction=viewrecord&id=L2032843231&from=export 10.1136/bmjgh-2023-014737 PMC11168176 38857944

[79] Motlogeloa O, Fitchett JM. Assessing the impact of climatic variability on acute respiratory diseases across diverse climatic zones in South Africa. Science of the Total Environment [Internet]. 2024;918((Motlogeloa O.; Fitchett J.M., Jennifer.Fitchett@wits.ac.za) School of Geography, Archaeology and Environmental Studies, University of the Witwatersrand, Johannesburg, South Africa). Available from: https://www.embase.com/search/results?subaction=viewrecord&id=L2030310632&from=export 10.1016/j.scitotenv.2024.170661 38320698

[80] Moodley Y, Asare K, Tanser F, Tomita A. Maternal exposure to heat and its association with miscarriage in rural KwaZulu-Natal, South Africa: A population-based cohort study. Women’s Health [Internet]. 2024;20((Moodley Y., yoshanm@sun.ac.za; Tanser F.) Africa Health Research Institute, KwaZulu-Natal, South Africa(Moodley Y., yoshanm@sun.ac.za) Faculty of Health and Environmental Sciences, Central University of Technology, Bloemfontein, South Africa(Moodley Y.,). Available from: https://www.embase.com/search/results?subaction=viewrecord&id=L2030744114&from=export 10.1177/17455057241259171 PMC11282531 39066467

[81] Powers JE, Mureithi M, Mboya J, Campolo J, Swarthout JM, Pajka J, et al. Effects of high temperature and heavy precipitation on drinking water quality and child hand contamination levels in rural kenya. bioRxiv [Internet]. 2022;((Powers J.E.; Pickering A.J., pickering@berkeley.edu) University of California, Berkeley, United States(Mureithi M.; Mboya J.) Innovations for Poverty Action, United States(Campolo J.) Farmers Business Network, United States(Swarthout J.M.; Pajka J.) Tuft). Available from: https://www.embase.com/search/results?subaction=viewrecord&id=L2021262276&from=export

[82] Longbottom J, Lord J, Torr S. Assessing the impact of climate change on sleeping sickness in zimbabwe using a geospatial mo Del of tsetse population dynamics. Transactions of the Royal Society of Tropical Medicine and Hygiene [Internet]. 2019;113((Longbottom J.; Lord J.; Torr S.) Dept. of Vector Biology, Liverpool School of Tropical Medicine, Liverpool, United Kingdom(Longbottom J.) Centre for Health Informatics,Computing and Statistics, Lancaster University, Lancaster, United Kingdom):S18–9. Available from: https://www.embase.com/search/results?subaction=viewrecord&id=L630553285&from=export

[83] Noureldin E, Shaffer L. Role of climatic factors in the incidence of dengue in port sudan city, sudan. Eastern Mediterranean Health Journal [Internet]. 2019;25(12):852–60. Available from: https://www.embase.com/search/results?subaction=viewrecord&id=L2003459721&from=export 32003443 10.26719/emhj.19.019

[84] Scovronick N, Sera F, Acquaotta F, Garzena D, Fratianni S, Wright CY, et al. The association between ambient temperature and mortality in South Africa: A time-series analysis. Environmental Research [Internet]. 2018;161((Scovronick N., Noah.Scovronick@princeton.edu) Woodrow Wilson School, Princeton University, Princeton, NJ, United States(Sera F.; Gasparrini A.) Department of Social and Environmental Health Research, London School of Hygiene and Tropical Medicine, London):229–35. Available from: https://www.embase.com/search/results?subaction=viewrecord&id=L619359144&from=export 10.1016/j.envres.2017.11.001 PMC5773242 29161655

[85] Singer T, Moore F, Luby S. Potential effects of increased global temperatures on neurological development factors in children under 5 years in east Africa: A modelling study. The Lancet Global Health [Internet]. 2016;4((Singer T., tgsinger@stanford.edu; Moore F.; Luby S.) Emmett Interdisciplinary Program for Environment and Resources, Y2E2 Building, 473 Via Ortega, Stanford, CA, United States(Singer T., tgsinger@stanford.edu; Luby S.) Stanford University, School of Medi):21. Available from: https://www.embase.com/search/results?subaction=viewrecord&id=L613443189&from=export

[86] Oloukoi G, Bob U, Jaggernath J. Perception and trends of associated health risks with seasonal climate variation in Oke-Ogun region, Nigeria. Health and Place [Internet]. 2014;25((Oloukoi G., oreofeadeniji@yahoo.com) Department of Environmental Management, Lead City University, Box 30678, Ibadan, Nigeria(Bob U., bobu@ukzn.ac.za; Jaggernath J., 200000983@ukzn.ac.za) School of Environmental Studies, University of Kwazulu-Natal, Sout):47–55. Available from: https://www.embase.com/search/results?subaction=viewrecord&id=L370306851&from=export 10.1016/j.healthplace.2013.09.009 24239703

[87] IHACC Research Team, Labbé J, Ford JD, Berrang-Ford L, Donnelly B, Lwasa S, et al. Vulnerability to the health effects of climate variability in rural southwestern Uganda. Mitig Adapt Strateg Glob Change [Internet]. 2016 Aug [cited 2023 Oct 15];21(6):931–53. Available from: http://link.springer.com/10.1007/s11027-015-9635-2

[88] Chapman S, Birch CE, Marsham JH, Part C, Hajat S, Chersich MF, et al. Past and projected climate change impacts on heat-related child mortality in Africa. Environ Res Lett. 2022;17(7):074028. doi: 10.1088/1748-9326/ac7ac5

[89] Fotso-Nguemo TC, Weber T, Diedhiou A, Chouto S, Vondou DA, Rechid D, et al. Projected Impact of Increased Global Warming on Heat Stress and Exposed Population Over Africa. Earth’s Futur [Internet]. 2023;11(1). Available from: https://www.scopus.com/inward/record.uri?eid=2-s2.0-85147154352&doi=10.1029%2f2022EF003268&partnerID=40&md5=921c960556a84d61289679e9526f9298

[90] Ouattara CA, Traore TI, Traore S, Sangare I, Meda CZ, Savadogo LGB. Climate factors and dengue fever in Burkina Faso from 2017 to 2019. J Public Health Afr [Internet]. 2022;13(1). Available from: https://www.scopus.com/inward/record.uri?eid=2-s2.0-85131358761&doi=10.4081%2fjphia.2022.2145&partnerID=40&md5=1e886b8d2def14352acc78899ff4abd4 10.4081/jphia.2022.2145 PMC9202460 35720791

[91] Abdullahi T, Nitschke G, Sweijd N. Predicting diarrhoea outbreaks with climate change. PLoS ONE [Internet]. 2022;17(4 April). Available from: https://www.scopus.com/inward/record.uri?eid=2-s2.0-85128643770&doi=10.1371%2fjournal.pone.0262008&partnerID=40&md5=199041f699e5d4c86f7b21713f74ff0e 10.1371/journal.pone.0262008 PMC9017952 35439258

[92] Frimpong K, Odonkor ST, Kuranchie FA, Nunfam VF. Evaluation of heat stress impacts and adaptations: perspectives from smallholder rural farmers in Bawku East of Northern Ghana. Heliyon [Internet]. 2020;6(4). Available from: https://www.scopus.com/inward/record.uri?eid=2-s2.0-85083301611&doi=10.1016%2fj.heliyon.2020.e03679&partnerID=40&md5=d40526b7e6b8f97ac196af8f43add50c 10.1016/j.heliyon.2020.e03679 PMC7177031 32337378

[93] Ikeda T, Kapwata T, Behera SK, Minakawa N, Hashizume M, Sweijd N, et al. Climatic factors in relation to diarrhoea hospital admissions in rural Limpopo, South Africa. Atmosphere [Internet]. 2019;10(9). Available from: https://www.scopus.com/inward/record.uri?eid=2-s2.0-85072259778&doi=10.3390%2fatmos10090522&partnerID=40&md5=33dd978533fd6e3e899da6b683e2d778

[94] Ncube A, Tawodzera M. Communities’ perceptions of health hazards induced by climate change in Mount Darwin district, Zimbabwe. Jamba J Disaster Risk Stud [Internet]. 2019;11(1):1–11. Available from: https://www.scopus.com/inward/record.uri?eid=2-s2.0-85110581268&doi=10.4102%2fJAMBA.V11I1.748&partnerID=40&md5=4b612ad43d64e602a5ff0ff83a5c42c7 10.4102/jamba.v11i1.748 PMC6620486 31308881

[95] Munyuli MT, Kavuvu J-MM, Mulinganya G, Bwinja GM. The Potential Financial Costs of Climate Change on Health of Urban and Rural Citizens: A Case Study of Vibrio cholerae Infections at Bukavu Town, South Kivu Province, Eastern of Democratic Republic of Congo. Iran J Public Health. 2013;42(7):707–25. 24427750 PMC3881617

[96] Lokotola CL, Wright CY, Wichmann J. Temperature as a modifier of the effects of air pollution on cardiovascular disease hospital admissions in Cape Town, South Africa. Environ Sci Pollut Res Int. 2020;27(14):16677–85. doi: 10.1007/s11356-020-07938-7 32133609

[97] Beyeler NS, Nicastro TM, Jawuoro S, Odhiambo G, Whittle HJ, Bukusi EA, et al. Pathways from climate change to emotional wellbeing: A qualitative study of Kenyan smallholder farmers living with HIV. PLOS Glob Public Health. 2023;3(7):e0002152. doi: 10.1371/journal.pgph.0002152 37490427 PMC10368256

[98] Cheruiyot SJ, Kimanthi M, Shabani JS, Nyamu NF, Gathu C, Agoi F, et al. Climate change poses a threat to nutrition and food security in Kilifi County, Kenya. Afr j prim health care fam med [Internet]. 2022 Oct 31 [cited 2023 Oct 15];14(1). Available from: https://phcfm.org/index.php/phcfm/article/view/3718 10.4102/phcfm.v14i1.3718 PMC9634675 36331200

[99] Nilsson M, Sie A, Muindi K, Bunker A, Ingole V, Ebi KL. Weather, climate, and climate change research to protect human health in sub-Saharan Africa and South Asia. Global Health Action [Internet]. 2021 Oct 26 [cited 2023 Oct 15];14(sup1):1984014. Available from: https://www.tandfonline.com/doi/full/10.1080/16549716.2021.1984014 10.1080/16549716.2021.1984014 PMC8986241 35377292

[100] Scheerens C, Bekaert E, Ray S, Essuman A, Mash B, Decat P, et al. Family Physician Perceptions of Climate Change, Migration, Health, and Healthcare in Sub-Saharan Africa: An Exploratory Study. IJERPH [Internet]. 2021 Jun 11 [cited 2023 Oct 15];18(12):6323. Available from: https://www.mdpi.com/1660-4601/18/12/6323 10.3390/ijerph18126323 PMC8296126 34207979

[101] Shezi B, Mathee A, Siziba W, Street RA, Naicker N, Kunene Z, et al. Environmental health practitioners potentially play a key role in helping communities adapt to climate change. BMC Public Health. 2019;19(1):54. doi: 10.1186/s12889-018-6378-5 30634954 PMC6330385

[102] Barimah AJ, Abdul-Ganiyu M, Ibrahim MM, Allotey SS, Commey RD, Osei-Tutu AG, et al. Investigating health professionals’ understanding and risk perception of the effect of climate change on health. A cross-sectional study of health professionals at the sda hospital, regional hospital and municipal hospital-Sunyani, Ghana. medRxiv [Internet]. 2023;((Barimah A.J., Quabenabarimah89@yahoo.com; Ibrahim M.M., Mimkp2004@yahoo.com; Allotey S.S., Sak_allot@yahoo.com; Commey R.D., Beccacemay1@gmail.com; Nketiah Y.B., Boakyenana14@gmail.com; Amoah B.O., bopokuamoah@gmail.com; Agyemang L., Laray0111@gmail.com;). Available from: https://www.embase.com/search/results?subaction=viewrecord&id=L2024975304&from=export

[103] Ofulla AVO, Gichere SK, Olado GO, Abuom PO, Anyona DN, Othero DM, et al. Effects of regional climate variability on the prevalence of diseases and their economic impacts on households in the Lake Victoria basin of Western Kenya. IJGW. 2016;10(1/2/3):332. doi: 10.1504/ijgw.2016.077899

[104] Girotto CD, Behzadian K, Musah A, Chen AS, Djordjević S, Nichols G, et al. Analysis of environmental factors influencing endemic cholera risks in sub-Saharan Africa. Sci Total Environ. 2024;926:171896. doi: 10.1016/j.scitotenv.2024.171896 38522541

[105] Guo D, Thomas J, Lazaro AB, Matwewe F, Johnson F. Modelling the influence of short-term climate variability on drinking water quality in tropical developing countries: A case study in Tanzania. Science of The Total Environment [Internet]. 2021 Apr [cited 2023 Dec 22];763:142932. Available from: https://linkinghub.elsevier.com/retrieve/pii/S0048969720364627 10.1016/j.scitotenv.2020.142932 33268262

[106] Horn LM, Hajat A, Sheppard L, Quinn C, Colborn J, Zermoglio MF, et al. Association between Precipitation and Diarrheal Disease in Mozambique. Int J Environ Res Public Health. 2018;15(4):709. doi: 10.3390/ijerph15040709 29642611 PMC5923751

[107] Olutumise AI, Ajibefun IA, Omonijo AG. Effect of climate variability on healthcare expenditure of food crop farmers in Southwest, Nigeria. Int J Biometeorol. 2021;65(6):951–61. doi: 10.1007/s00484-021-02079-z 33474613

[108] Arabi M. The effect of climatic factors on cholera incidence in the far north region of Cameroon. American Journal of Tropical Medicine and Hygiene [Internet]. 2013;89(5):207. Available from: https://www.embase.com/search/results?subaction=viewrecord&id=L71312514&from=export

[109] Constantin de Magny G, Thiaw W, Kumar V, Manga NM, Diop BM, Gueye L, et al. Cholera Outbreak in Senegal in 2005: Was Climate a Factor? PLoS ONE [Internet]. 2012;7(8). Available from: https://www.embase.com/search/results?subaction=viewrecord&id=L365552122&from=export 10.1371/journal.pone.0044577 PMC3432123 22952995

[110] Uttajug A, Ueda K, Seposo X, Francis JM. Association between extreme rainfall and acute respiratory infection among children under-5 years in sub-Saharan Africa: an analysis of Demographic and Health Survey data, 2006-2020. BMJ Open. 2023;13(4):e071874. doi: 10.1136/bmjopen-2023-071874 37185183 PMC10152048

[111] Yeboah E, Kuunibe N, Mank I, Parisi D, Bonnet E, Lohmann J, et al. Every drop matters: Combining population-based and satellite data to investigate the link between lifetime rainfall exposure and chronic undernutrition in children under five years in rural Burkina Faso. EnvironResLett [Internet]. 2022;17(5). Available from: https://www.scopus.com/inward/record.uri?eid=2-s2.0-85130611915&doi=10.1088%2f1748-9326%2fac661c&partnerID=40&md5=7147597192dd762edc44a23cd3147639

[112] Thiam S, Diène AN, Sy I, Winkler MS, Schindler C, Ndione JA, et al. Association between Childhood Diarrhoeal Incidence and Climatic Factors in Urban and Rural Settings in the Health District of Mbour, Senegal. Int J Environ Res Public Health. 2017;14(9):1049. doi: 10.3390/ijerph14091049 28895927 PMC5615586

[113] Epstein A, Benmarhnia T, Weiser SD. Drought and Illness among Young Children in Uganda, 2009–2012. The American Journal of Tropical Medicine and Hygiene [Internet]. 2020 Mar 5 [cited 2023 Dec 22];102(3):644–8. Available from: https://ajtmh.org/doi/10.4269/ajtmh.19-0412 31933457 10.4269/ajtmh.19-0412 PMC7056439

[114] Albers L, van Roosmalen J, Tura AK. Climate change and neonatal survival: the case of Ethiopia. Lancet Glob Health. 2016;4(4):e236. doi: 10.1016/S2214-109X(16)00045-0 27013308

[115] Amondo EI, Kirui OK, Mirzabaev A. Health gender gap in Uganda: do weather effects and water play a role? Int J Equity Health [Internet]. 2022 Dec 5 [cited 2023 Dec 22];21(1):173. Available from: https://equityhealthj.biomedcentral.com/articles/10.1186/s12939-022-01769-3 10.1186/s12939-022-01769-3 PMC9720924 36471369

[116] Bailey KM, McCleery RA, Barnes G, McKune SL. Climate-Driven Adaptation, Household Capital, and Nutritional Outcomes among Farmers in Eswatini. International Journal of Environmental Research and Public Health. 2019;16(21). 10.3390/ijerph16214063 PMC6862074 31652699

[117] Blackwell PJ. East Africa’s pastoralist emergency: is climate change the straw that breaks the camel’s back?. Third World Q. 2010;31(8):1321–38. doi: 10.1080/01436597.2010.541085 21506297

[118] Bryson JM, Patterson K, Berrang-Ford L, Lwasa S, Namanya DB, Twesigomwe S, et al. Seasonality, climate change, and food security during pregnancy among Indigenous and non-Indigenous women in rural Uganda: Implications for maternal-infant health. PLoS One. 2021;16(3):e0247198. doi: 10.1371/journal.pone.0247198 33760848 PMC7990176

[119] Hadida G, Ali Z, Kastner T, Carr TW, Prentice AM, Green R, et al. Changes in Climate Vulnerability and Projected Water Stress of The Gambia’s Food Supply Between 1988 and 2018: Trading With Trade-Offs. Front Public Health. 2022;10:786071. doi: 10.3389/fpubh.2022.786071 35747777 PMC9211751

[120] Hagos S, Lunde T, Mariam DH, Woldehanna T, Lindtjørn B. Climate change, crop production and child under nutrition in Ethiopia; a longitudinal panel study. BMC Public Health. 2014;14:884. doi: 10.1186/1471-2458-14-884 25163522 PMC4158109

[121] Kemajou Njatang D, Bouba Djourdebbé D, Adda Wadou ND. Climate variability, armed conflicts and child malnutrition in sub-saharan Africa: A spatial analysis in Ethiopia, Kenya and Nigeria. Heliyon. 2023;9(11):e21672. 10.1016/j.heliyon.2023.e21672 PMC10656247 38027550

[122] Lee TT, Dalvie MA, Röösli M, Merten S, Kwiatkowski M, Mahomed H, et al. Understanding diarrhoeal diseases in response to climate variability and drought in Cape Town, South Africa: a mixed methods approach. Infect Dis Poverty [Internet]. 2023 Aug 18 [cited 2023 Dec 22];12(1):76. Available from: https://idpjournal.biomedcentral.com/articles/10.1186/s40249-023-01127-7 10.1186/s40249-023-01127-7 PMC10436439 37596648

[123] Lindvall K, Kinsman J, Abraha A, Dalmar A, Abdullahi MF, Godefay H, et al. Health Status and Health Care Needs of Drought-Related Migrants in the Horn of Africa-A Qualitative Investigation. Int J Environ Res Public Health. 2020;17(16):5917. doi: 10.3390/ijerph17165917 32824046 PMC7459765

[124] Rawat A, Karlstrom J, Ameha A, Oulare M, Omer MD, Desta HH, et al. The contribution of community health systems to resilience: Case study of the response to the drought in Ethiopia. J Glob Health. 2022;12:14001. doi: 10.7189/jogh.12.14001 36273279 PMC9588157

[125] Rosen JG, Mulenga D, Phiri L, Okpara N, Brander C, Chelwa N, et al. “Burnt by the scorching sun”: climate-induced livelihood transformations, reproductive health, and fertility trajectories in drought-affected communities of Zambia. BMC Public Health [Internet]. 2021 Dec [cited 2023 Oct 15];21(1):1501. Available from: https://bmcpublichealth.biomedcentral.com/articles/10.1186/s12889-021-11560-8 10.1186/s12889-021-11560-8 PMC8335992 34344335

[126] Nyadzayo T, Zvanaka S, Kanyowa T, Mathieu J, Kambarami T, Nemaramba M, et al. Enhancing capacity of Zimbabwe’s health system to respond to climate change induced drought: a rapid nutritional assessment. Pan Afr Med J [Internet]. 2021 [cited 2023 Dec 22];40. Available from: https://www.panafrican-med-journal.com/content/article/40/113/full 10.11604/pamj.2021.40.113.30545 PMC8627146 34887987

[127] Sorgho R, Mank I, Kagoné M, Souares A, Danquah I, Sauerborn R. “We Will Always Ask Ourselves the Question of How to Feed the Family”: Subsistence Farmers’ Perceptions on Adaptation to Climate Change in Burkina Faso. Int J Environ Res Public Health. 2020;17(19):7200. doi: 10.3390/ijerph17197200 33019715 PMC7579300

[128] Walton S, Jessani NS, Jue-Wong H, Hazel EA, Akseer N, Kante AM, et al. Climate shocks and nutrition: The role of food security policies and programs in enhancing maternal and neonatal survival in Niger. Matern Child Nutr. 2024;20(1):e13566. doi: 10.1111/mcn.13566 37794716 PMC10750024

[129] Treibich C, Bell E, Lépine A, Blanc E. From a drought to HIV: An analysis of the effect of droughts on transactional sex and sexually transmitted infections in Malawi. SSM - Population Health [Internet]. 2022;19((Treibich C., carole.treibich@univ-grenoble-alpes.fr) Univ. Grenoble Alpes, CNRS, INRAE, GAEL, Grenoble INP, Grenoble, France(Bell E., ebell@ohe.org) Office of Health Economics, United Kingdom(Lépine A., a.lepine@ucl.ac.uk) Institute for Global Health, Un). Available from: https://www.embase.com/search/results?subaction=viewrecord&id=L2020289742&from=export 10.1016/j.ssmph.2022.101221 PMC9508466 36164494

[130] Epstein A, Bendavid E, Nash D, Charlebois ED, Weiser SD. Drought and intimate partner violence towards women in 19 countries in sub-saharan Africa during 2011-2018: A population-based study. PLoS Medicine [Internet]. 2020;17(3). Available from: https://www.embase.com/search/results?subaction=viewrecord&id=L2007311787&from=export 10.1371/journal.pmed.1003064 PMC7081984 32191701

[131] Low AJ, Frederix K, McCracken S, Manyau S, Gummerson E, Radin E, et al. Association between severe drought and HIV prevention and care behaviors in Lesotho: A population-based survey 2016-2017. PLoS Medicine [Internet]. 2019;16(1). Available from: https://www.embase.com/search/results?subaction=viewrecord&id=L2001519362&from=export 10.1371/journal.pmed.1002727 PMC6331084 30640916

[132] Bahru BA, Bosch C, Birner R, Zeller M. Drought and child undernutrition in Ethiopia: A longitudinal path analysis. PLoS ONE [Internet]. 2019;14(6). Available from: https://www.scopus.com/inward/record.uri?eid=2-s2.0-85067297408&doi=10.1371%2fjournal.pone.0217821&partnerID=40&md5=e8e06a56c103f8ab30e4e3f2efe67683 10.1371/journal.pone.0217821 PMC6576771 31206545

[133] Abu M, Heath SC, Adger WN, Codjoe SNA, Butler C, Quinn T. Social consequences of planned relocation in response to sea level rise: impacts on anxiety, well-being, and perceived safety. Sci Rep. 2024;14(1):3461. doi: 10.1038/s41598-024-53277-9 38342949 PMC10859369

[134] Adams EA, Nyantakyi-Frimpong H. Stressed, anxious, and sick from the floods: A photovoice study of climate extremes, differentiated vulnerabilities, and health in Old Fadama, Accra, Ghana. Health & Place [Internet]. 2021 Jan [cited 2023 Dec 22];67:102500. Available from: https://linkinghub.elsevier.com/retrieve/pii/S1353829220318943 10.1016/j.healthplace.2020.102500 33373811

[135] Alexander KA, Heaney AK, Shaman J. Hydrometeorology and flood pulse dynamics drive diarrheal disease outbreaks and increase vulnerability to climate change in surface-water-dependent populations: A retrospective analysis. Patz JA, editor. PLoS Med [Internet]. 2018 Nov 8 [cited 2023 Dec 22];15(11):e1002688. Available from: https://dx.plos.org/10.1371/journal.pmed.1002688 10.1371/journal.pmed.1002688 PMC6224043 30408029

[136] Nöthling J, Gibbs A, Washington L, Gigaba SG, Willan S, Abrahams N, et al. Change in emotional distress, anxiety, depression and PTSD from pre- to post-flood exposure in women residing in low-income settings in South Africa. Arch Women’s Ment Health [Internet]. 2024;27(2):201–18. Available from: https://www.scopus.com/inward/record.uri?eid=2-s2.0-85177603481&doi=10.1007%2fs00737-023-01384-3&partnerID=40&md5=534e31c022812588d66d917cff14818c 37989799 10.1007/s00737-023-01384-3 PMC10933147

[137] Connolly K, Mbutu M, Bartram J, Fuente D. Perceptions of climate-related risk among water sector professionals in Africa-Insights from the 2016 African Water Association Congress. Int J Hyg Environ Health. 2018;221(5):838–46. doi: 10.1016/j.ijheh.2018.04.007 29853293

[138] Dovie DBK, Dzodzomenyo M, Ogunseitan OA. Sensitivity of health sector indicators’ response to climate change in Ghana. Sci Total Environ. 2017;574:837–46. doi: 10.1016/j.scitotenv.2016.09.066 27665444

[139] Greibe Andersen J, Karekezi C, Ali Z, Yonga G, Kallestrup P, Kraef C. Perspectives of Local Community Leaders, Health Care Workers, Volunteers, Policy Makers and Academia on Climate Change Related Health Risks in Mukuru Informal Settlement in Nairobi, Kenya-A Qualitative Study. Int J Environ Res Public Health. 2021;18(22):12241. doi: 10.3390/ijerph182212241 34831995 PMC8618671

[140] Kapwata T, Kunene Z, Wernecke B, Lange S, Howard G, Nijhawan A, et al. Applying a WASH Risk Assessment Tool in a Rural South African Setting to Identify Risks and Opportunities for Climate Resilient Communities. IJERPH [Internet]. 2022 Feb 25 [cited 2023 Dec 22];19(5):2664. Available from: https://www.mdpi.com/1660-4601/19/5/2664 10.3390/ijerph19052664 PMC8909929 35270357

[141] Kemajou DN. Climate variability, water supply, sanitation and diarrhea among children under five in Sub-Saharan Africa: a multilevel analysis. Journal of Water and Health [Internet]. 2022 Apr 1 [cited 2023 Oct 15];20(4):589–600. Available from: https://iwaponline.com/jwh/article/20/4/589/88132/Climate-variability-water-supply-sanitation-and 10.2166/wh.2022.199 35482376

[142] Mabhaudhi T, Nhamo L, Mpandeli S, Nhemachena C, Senzanje A, Sobratee N, et al. The Water-Energy-Food Nexus as a Tool to Transform Rural Livelihoods and Well-Being in Southern Africa. Int J Environ Res Public Health. 2019;16(16):2970. doi: 10.3390/ijerph16162970 31426610 PMC6720849

[143] McCreesh N, Nikulin G, Booth M. Predicting the effects of climate change on Schistosoma mansoni transmission in eastern Africa. Parasit Vectors [Internet]. 2015 [cited 2023 Oct 15];8(1):4. Available from: http://www.parasitesandvectors.com/content/8/1/4 10.1186/s13071-014-0617-0 PMC4297451 25558917

[144] Powers JE, Mureithi M, Mboya J, Campolo J, Swarthout JM, Pajka J, et al. Effects of High Temperature and Heavy Precipitation on Drinking Water Quality and Child Hand Contamination Levels in Rural Kenya. Environ Sci Technol. 2023;57(17):6975–88. doi: 10.1021/acs.est.2c07284 37071701 PMC10157894

[145] Rankoana SA. Climate change impacts on indigenous health promotion: the case study of Dikgale community in Limpopo Province, South Africa. Glob Health Promot [Internet]. 2022 Mar [cited 2023 Dec 22];29(1):58–64. Available from: http://journals.sagepub.com/doi/10.1177/17579759211015183 10.1177/17579759211015183 34109875

[146] Van Den Berg H, Rickert B, Ibrahim S, Bekure K, Gichile H, Girma S, et al. Linking water quality monitoring and climate-resilient water safety planning in two urban drinking water utilities in Ethiopia. Journal of Water and Health [Internet]. 2019 Dec 1 [cited 2023 Dec 22];17(6):989–1001. Available from: https://iwaponline.com/jwh/article/17/6/989/70432/Linking-water-quality-monitoring-and 10.2166/wh.2019.059 31850905

[147] Sheriff M, Mash R. Climate change and primary health care in Chakama, Kilifi County, Kenya. Afr j prim health care fam med [Internet]. 2022 Sep 27 [cited 2023 Dec 22];14(1). Available from: https://phcfm.org/index.php/phcfm/article/view/3670 10.4102/phcfm.v14i1.3670 PMC9575365 36226937

[148] Nwankwo ONO, Nwankwo GI, Akpa C, Anyigor CA, Akpoke MA, Onwe F. Perceptions and experiences of physicians on the health effects of climate change in a Vulnearable City in Africa. American Journal of Respiratory and Critical Care Medicine [Internet]. 2017;195((Nwankwo O.N.O., drogonnanwankwo@gmail.com) University of Calabar, Teaching Hospital, Calabar, Nigeria(Nwankwo G.I.; Akpa C.; Anyigor C.A.) Federal Teaching Hospital, Abakaliki, Nigeria(Akpoke M.A.) AMURT health services, Abakaliki, Nigeria(Onwe F.) State). Available from: https://www.embase.com/search/results?subaction=viewrecord&id=L617722930&from=export

[149] Reyburn R, Kim DR, Emch M, Khatib A, Von Seidlein L, Ali M. Climate variability and the outbreaks of cholera in Zanzibar, East Africa: A time series analysis. American Journal of Tropical Medicine and Hygiene [Internet]. 2011;84(6):862–9. Available from: https://www.embase.com/search/results?subaction=viewrecord&id=L361938561&from=export 21633020 10.4269/ajtmh.2011.10-0277 PMC3110353

[150] Mutuku FM, King CH, Bustinduy AL, Mungai PL, Muchiri EM, Kitron U. Impact of drought on the spatial pattern of transmission of Schistosoma haematobium in coastal Kenya. American Journal of Tropical Medicine and Hygiene [Internet]. 2011;85(6):1065–70. Available from: https://www.embase.com/search/results?subaction=viewrecord&id=L363069081&from=export 22144445 10.4269/ajtmh.2011.11-0186 PMC3225153

[151] Libanda B, Rand E, Gyang GN, Sindano CT, Simwanza L, Chongo M. Recent and future exposure of water, sanitation, and hygiene systems to climate-related hazards in Zambia. J Water Clim Change [Internet]. 2024;15(3):958–77. Available from: https://www.scopus.com/inward/record.uri?eid=2-s2.0-85189858223&doi=10.2166%2fwcc.2024.392&partnerID=40&md5=eee892781177036668685c90956a3778

[152] Cecchi G, Courtin F, Paone M, Diarra A, Franco JR, Mattioli RC, et al. Mapping sleeping sickness in Western Africa in a context of demographic transition and climate change. Parasite. 2009;16(2):99–106. doi: 10.1051/parasite/2009162099 19585887

[153] Greibe Andersen J, Kallestrup P, Karekezi C, Yonga G, Kraef C. Climate change and health risks in Mukuru informal settlement in Nairobi, Kenya – knowledge, attitudes and practices among residents. BMC Public Health [Internet]. 2023 Feb 25 [cited 2023 Oct 15];23(1):393. Available from: https://bmcpublichealth.biomedcentral.com/articles/10.1186/s12889-023-15281-y 10.1186/s12889-023-15281-y PMC9958313 36841782

[154] Hongoh V, Michel P, Gosselin P, Samoura K, Ravel A, Campagna C, et al. Multi-Stakeholder Decision Aid for Improved Prioritization of the Public Health Impact of Climate Sensitive Infectious Diseases. Int J Environ Res Public Health. 2016;13(4):419. doi: 10.3390/ijerph13040419 27077875 PMC4847081

[155] John L, Shekede MD, Gwitira I, Mazhindu AN, Pfukenyi DM, Chikerema S. Modelling climate change impacts on the spatial distribution of anthrax in Zimbabwe. BMC Public Health. 2024;24(1):632. doi: 10.1186/s12889-024-17856-9 38418986 PMC10900681

[156] Kimaro EG, Toribio J-ALML, Mor SM. Climate change and cattle vector-borne diseases: Use of participatory epidemiology to investigate experiences in pastoral communities in Northern Tanzania. Prev Vet Med. 2017;147:79–89. doi: 10.1016/j.prevetmed.2017.08.010 29254730

[157] Otieno FT, Gachohi J, Gikuma-Njuru P, Kariuki P, Oyas H, Canfield SA, et al. Modeling the Potential Future Distribution of Anthrax Outbreaks under Multiple Climate Change Scenarios for Kenya. Int J Environ Res Public Health. 2021;18(8):4176. doi: 10.3390/ijerph18084176 33920863 PMC8103515

[158] Tabo Z, Kalinda C, Breuer L, Albrecht C. Exploring the interplay between climate change and schistosomiasis transmission dynamics. Infect Dis Model. 2023;9(1):158–76. doi: 10.1016/j.idm.2023.12.003 38268699 PMC10805680

[159] Taylor D, Hagenlocher M, Jones AE, Kienberger S, Leedale J, Morse AP. Environmental change and Rift Valley fever in eastern Africa: projecting beyond HEALTHY FUTURES. Geospat Health [Internet]. 2016 Mar 31 [cited 2023 Dec 22];11(1s). Available from: http://www.geospatialhealth.net/index.php/gh/article/view/387 10.4081/gh.2016.387 27063733

[160] Abdullahi B, Mutiso J, Maloba F, Macharia J, Riongoita M, Gicheru M. Climate change and environmental influence on visceral leishmaniasis in West Pokot county, Kenya. medRxiv [Internet]. 2022;((Abdullahi B., bulle07@hotmail.co.uk) Department of Community Health and Epidemiology, School of Public Health, Kenyatta University, Nairobi, Kenya(Mutiso J.; Maloba F.; Macharia J.; Gicheru M.) Department of Zoological Science, School of Pure and Applied). Available from: https://www.embase.com/search/results?subaction=viewrecord&id=L2018694198&from=export

[161] Thomassen HA, Fuller T, Asefi-Najafabady S, Shiplacoff JAG, Mulembakani PM, Blumberg S, et al. Pathogen-Host Associations and Predicted Range Shifts of Human Monkeypox in Response to Climate Change in Central Africa. PLoS ONE [Internet]. 2013;8(7). Available from: https://www.embase.com/search/results?subaction=viewrecord&id=L369507681&from=export 10.1371/journal.pone.0066071 PMC3729955 23935820

[162] Agbossou A, Fontodji JK, Ayassou K, Tchegueni S, Segla KN, Adjonou K, et al. Integrated climate change and air pollution mitigation assessment for Togo. Sci Total Environ. 2022;844:157107. doi: 10.1016/j.scitotenv.2022.157107 35810891

[163] Miyayo SF, Owili PO, Muga MA, Lin T-H. Analysis of Pneumonia Occurrence in Relation to Climate Change in Tanga, Tanzania. Int J Environ Res Public Health. 2021;18(9):4731. doi: 10.3390/ijerph18094731 33946714 PMC8125699

[164] Nyoka R, Omony J, Mwalili SM, Achia TNO, Gichangi A, Mwambi H. Effect of climate on incidence of respiratory syncytial virus infections in a refugee camp in Kenya: A non-Gaussian time-series analysis. PLoS ONE [Internet]. 2017;12(6). Available from: https://www.embase.com/search/results?subaction=viewrecord&id=L616529571&from=export 10.1371/journal.pone.0178323 PMC5453485 28570627

[165] Lubinda J, Haque U, Bi Y, Shad MY, Keellings D, Hamainza B, et al. Climate change and the dynamics of age-related malaria incidence in Southern Africa. Environmental Research [Internet]. 2021 Jun [cited 2023 Dec 22];197:111017. Available from: https://linkinghub.elsevier.com/retrieve/pii/S001393512100311X 10.1016/j.envres.2021.111017 33766570

[166] Kadio K, Filippi V, Congo M, Scorgie F, Roos N, Lusambili A, et al. Extreme heat, pregnancy and women’s well-being in Burkina Faso: an ethnographical study. BMJ Glob Health. 2024;8(Suppl 3):e014230. doi: 10.1136/bmjgh-2023-014230 38382997 PMC10897842

[167] Musengimana G, Mukinda FK, Machekano R, Mahomed H. Temperature variability and occurrence of diarrhoea in children under five-years-old in Cape Town metropolitan sub-districts. Int J Environ Res Public Health. 2016;13(9). 10.3390/ijerph13090859 PMC5036692 27589772

[168] Jennings S, Challinor A, Smith P, Macdiarmid JI, Pope E, Chapman S, et al. Stakeholder-driven transformative adaptation is needed for climate-smart nutrition security in sub-Saharan Africa. Nat Food. 2024;5(1):37–47. doi: 10.1038/s43016-023-00901-y 38168785 PMC10810754

[169] Rovin K, Hardee K, Kidanu A. Linking population, fertility, and family planning with adaptation to climate change: perspectives from Ethiopia. Afr J Reprod Health. 2013;17(3):15–29. 24069764

[170] Wright CY, Mathee A, Goldstone C, Naidoo N, Kapwata T, Wernecke B, et al. Developing a Healthy Environment Assessment Tool (HEAT) to Address Heat-Health Vulnerability in South African Towns in a Warming World. Int J Environ Res Public Health. 2023;20(4):2852. doi: 10.3390/ijerph20042852 36833550 PMC9957206

[171] Azeez RO, Rampedi IT, Ifegbesan AP, Ogunyemi B. Geo-demographics and source of information as determinants of climate change consciousness among citizens in African countries. Heliyon. 2024;10(7):e27872. doi: 10.1016/j.heliyon.2024.e27872 38560259 PMC10981018

[172] Sulser TB, Beach RH, Wiebe KD, Dunston S, Fukagawa NK. Disability-adjusted life years due to chronic and hidden hunger under food system evolution with climate change and adaptation to 2050. Am J Clin Nutr. 2021;114(2):550–63. doi: 10.1093/ajcn/nqab101 34013962 PMC8326044

[173] Nunfam VF, Oosthuizen J, Adusei-Asante K, Van Etten EJ, Frimpong K. Perceptions of climate change and occupational heat stress risks and adaptation strategies of mining workers in Ghana. Sci Total Environ. 2019;657:365–78. doi: 10.1016/j.scitotenv.2018.11.480 30550901

[174] Mpandeli S, Naidoo D, Mabhaudhi T, Nhemachena C, Nhamo L, Liphadzi S, et al. Climate Change Adaptation through the Water-Energy-Food Nexus in Southern Africa. Int J Environ Res Public Health. 2018;15(10):2306. doi: 10.3390/ijerph15102306 30347771 PMC6210720

[175] Henri Aurélien AB. Vulnerability to climate change in sub-Saharan Africa countries. Does international trade matter? Heliyon [Internet]. 2025 Feb 28 [cited 2025 Apr 25];11(4):e42517. Available from: https://www.sciencedirect.com/science/article/pii/S2405844025008977 10.1016/j.heliyon.2025.e42517 PMC11874565 40034296

[176] Williams J, Chin-Yee S, Maslin M, Barnsley J, Costello A, Lang J. Africa and climate justice at COP27 and beyond: impacts and solutions through an interdisciplinary lens. UCL Open Environ. 2025;5:e062. 10.14324/111.444/ucloe.000062 PMC10476578 37671394

[177] Seidler V, Utazi EC, Finaret AB, Luckeneder S, Zens G, Bodarenko M, et al. Subnational variations in the quality of household survey data in sub-Saharan Africa. Nat Commun. 2025;16(1):3771. doi: 10.1038/s41467-025-58776-5 40263256 PMC12015360

[178] Brand D, Singh JA, Nienaber McKay AG, Cengiz N, Moodley K. Data sharing governance in sub-Saharan Africa during public health emergencies: Gaps and guidance. S Afr J Sci [Internet]. 2022 [cited 2025 Apr 25];118(11–12):10.17159/sajs.2022/13892. Available from: https://www.ncbi.nlm.nih.gov/pmc/articles/PMC11241865/10.17159/sajs.2022/13892 PMC11241865 39005847

[179] CDC. About CDC. 2024 [cited 2024 Jun 19]. Social Determinants of Health (SDOH). Available from: https://www.cdc.gov/about/priorities/why-is-addressing-sdoh-important.html

[180] Rigon A. A review of intersectionality and climate change and the potential of intersectional participatory methods and storytelling to co-produce climate justice. Climate and Development. 2025;:1–13. doi: 10.1080/17565529.2025.2477105

[181] Feigin VL, Roth GA, Naghavi M, Parmar P, Krishnamurthi R, Chugh S, et al. Global burden of stroke and risk factors in 188 countries, during 1990-2013: a systematic analysis for the Global Burden of Disease Study 2013. Lancet Neurol. 2016;15(9):913–24. doi: 10.1016/S1474-4422(16)30073-4 27291521

[182] Wright CY, Kapwata T, Naidoo N, Asante KP, Arku RE, Cissé G, et al. Climate Change and Human Health in Africa in Relation to Opportunities to Strengthen Mitigating Potential and Adaptive Capacity: Strategies to Inform an African “Brains Trust”. Ann Glob Health. 2024;90(1):7. doi: 10.5334/aogh.4260 38312714 PMC10836170

[183] Quintana AV, Mayhew SH, Kovats S, Gilson L. A story of (in)coherence: climate adaptation for health in South African policies. Health Policy Plan. 2024;39(4):400–11. doi: 10.1093/heapol/czae011 38491988 PMC11005833

[184] Wright CY, Garland RM, Norval M, Vogel C. Human health impacts in a changing South African climate. S Afr Med J. 2014;104(8):579. 26307804 10.7196/samj.8603

