# Supplementary material for: Assessing Sub-Saharan Africa’s readiness to address the impact of climate change and health: A scoping review
Source: PLoS One. 2025 Nov 11;20(11):e0315482. doi: 10.1371/journal.pone.0315482 (PMC12604764; doi:10.1371/journal.pone.0315482)
Supplement: S1 Table — (DOCX) [file pone.0315482.s003.docx]

**Supplemental Information For: Assessing Sub-Saharan Africa's Readiness to Address the Impact of Climate Change and Health: A Scoping Review**

**Short Title: Climate Change and Health Sub-Saharan Africa**

Aminata Kilungo 1^1*^, God’sgift Chukwuonye^2^, Victor Okpanachi^1^, Hussein Mohamed^3^

^1^ Department of Community, Environment and Policy, University of Arizona, Tucson, Arizona

^2^ Department of Environmental Sciences, The University of Arizona, Tucson, Arizona

^3^ Department of Environmental and Occupational Health, Muhimbili University of Health and Allied Sciences, Dar es Salaam, Tanzania

*Corresponding Author:
Aminata Kilungo
Telephone: 520.626.8565
Email: [paminata@arizona.edu](mailto:paminata@arizona.edu)

S1 Fig. Vulnerable Groups Identified in Past Studies and the Number of times the group was mentioned.

S2 Fig. Adaptation Strategies Suggested by Authors in Current Study to Address Climate Change and Health Issues in Sub-Saharan Africa

**Tables**

S1 Table. Number of Studies on Climate Change and Health by Country in Sub-Saharan Africa

| Country | n | Studies |
| --- | --- | --- |
| Burkina Faso | 9 | (44,50,57,64,90,111,127,154,166) |
| Botswana | 2 | (1,135) |
| Kenya | 22 | (8,38,66,67,73,77,78,81,97,98,103,117,137,139,144,147,150,153,157,160,164,172) |
| South Africa | 24 | (4,10,14,18,31–33,35,53,62,63,76,79,80,84,93,96,101,136,140,145,160,167,170) |
| Mozambique | 2 | (16,106) |
| Tanzania | 10 | (20,42,69,72,78,85,105,149,156,163) |
| Uganda | 8 | (46,52,54,78,87,113,115,118) |
| Ghana | 14 | (2,37,41,56,59,61,70,71,92,102,133,134,138,173) |
| Nigeria | 5 | (34,48,86,107,148) |
| Cameroon | 1 | (108) |
| Zimbabwe | 6 | (45,65,82,94,126,155) |
| Ethiopia | 6 | (36,120,124,132,146,169) |
| DR Congo | 3 | (68,95,161) |
| Gambia | 3 | (55,74,119) |
| Eswatini | 1 | (116) |
| Zambia | 2 | (125,151) |
| Madagascar | 1 | (43) |
| Togo | 1 | (162) |
| Central Africa | 1 | (58) |
| Sudan | 1 | (83) |
| Senegal | 2 | (109,112) |
| Lesotho | 1 | (131) |
| Malawi | 1 | (129) |
| Rwanda | 1 | (49) |
| Study across multiple countries in SSA | 24 | (11,19,39,47,51,58,60,75,88,89,99,104,110,121,123,130,141–143,152,158,159,168,171) |
