# Supplementary material for: Assessing Sub-Saharan Africa’s readiness to address the impact of climate change and health: A scoping review
Source: PLoS One. 2025 Nov 11;20(11):e0315482. doi: 10.1371/journal.pone.0315482 (PMC12604764; doi:10.1371/journal.pone.0315482)
Supplement: S1 Checklist — (DOCX) [file pone.0315482.s004.docx]

| **Section and Topic** | **Item #** | **Checklist item** | **Location where item is reported** |
| --- | --- | --- | --- |
| **TITLE** | | |  |
| Title | 1 | Identify the report as a systematic review.  Preparedness of Sub-Saharan African Countries to Address Climate Change and Health Impact: A Scoping Review | Title page |
| **ABSTRACT** | | |  |
| Abstract | 2 | See the PRISMA 2020 for Abstracts checklist.  **Background:** Climate change severely threatens global public health, with sub-Saharan Africa (SSA) projected to experience profound impacts.  Objectives: to provide a comprehensive overview of current research on climate change and its health implications in SSA while identifying research gaps and outlining the necessary resources and policy interventions to strengthen public health resilience in the region.  **Study eligibility criteria:** Papers were included if they (a) addressed the conceptualization of climate change in sub-Saharan Africa, (b)  explored the impacts of climate change on health outcomes, (c) focused on relevant climate change impacts, including water quality, flooding, and drought and other health outcomes (d) were published between January 1, 2001, and August 1, 2024, and (d) were written in English.  **Information sources:** Literature was retrieved from Scopus, Embase, PubMed and Web of Science using the keywords “climate change,” “health,” and “sub-Saharan Africa”.  **Included studies:** 153 studies were included for review. The included studies were published between January 2001 and August 2024.  **Synthesis of results**: Although extensive studies have been conducted on extreme heat (71 studies), drought (45 studies), extreme precipitation events (52 studies), and flooding (34 studies), important themes such as air quality (10 studies), chemical water quality (8 studies) and natural disasters (8 studies), have been understudied. Additionally, this scoping review revealed a geographical gap in climate change and health studies, as only 24 out of 53 countries in sub-Saharan Africa were represented. The key deficiencies identified include limited funding, technological constraints, inadequate climate policies, and a lack of community-focused adaptation plans.  **Interpretation:** Moreover, this review highlights the urgent need for resilient healthcare systems capable of addressing climate-related health risks effectively. Addressing these gaps is essential for developing targeted strategies to mitigate climate change and its health impacts and increase resilience in SSA communities.  **Conclusion:** Strengthening research capacities, fostering collaboration, and implementing evidence-based policies are imperative steps toward achieving sustainable health outcomes in the face of a changing climate in sub-Saharan Africa. | 2 |
| **INTRODUCTION** | | |  |
| Rationale | 3 | Describe the rationale for the review in the context of existing knowledge.  Most research approach and plans are based on broader global perspectives. Given that sub-Saharan region has its unique challenges, research to guide adaptation, mitigation and resilience efforts must also be unique and region specific. Additionally, sub-Saharan Africa will be at the frontline of climate change. Therefore, it is important to explore the preparedness of this region to deal with current and future climate impacts. | 2, 3 |
| Objectives | 4 | Provide an explicit statement of the objective(s) or question(s) the review addresses.  The objective of this review is to examine current research on climate impacts and safety, health, policy, and to identify existing gaps in research and the need for policymaking to guide interventions to improve public health and build climate change resilience in Africa. The review addressed the following questions.   1. What are the geographic and thematic gaps in sub-Saharan Africa's climate change and health research, and how do these gaps affect our understanding of the region's climate-induced health outcomes and vulnerabilities? 2. How do extreme weather events, such as heatwaves, droughts, and floods, interact with social determinants of health to influence health vulnerabilities and outcomes in sub-Saharan Africa? 3. What practical solutions and community-based adaptation strategies can be developed and implemented to address air quality, water quality, and extreme heat, enhancing resilience, and mitigating health impacts in sub-Saharan Africa? | 2,3 |
| **METHODS** | | |  |
| Eligibility criteria | 5 | Specify the inclusion and exclusion criteria for the review and how studies were grouped for the syntheses.  **Studies were included if** (a) addressed the conceptualization of climate change in sub-Saharan Africa, (b) explored the impacts of climate change on human health outcomes, (c) focused on relevant climate change impacts, including water quality, flooding, and drought and other health outcomes (d) were published between January 1, 2001, and August 1, 2024, (e) were primary research, and (f) were written in English.  **Studies were excluded if** (a) they were not from the sub-Saharan Africa region (b) if they mentioned climate change impacts not relevant to the African context (e.g., changes in snowmelt or wildfires), (c) addressed an aspect of health nor related to humans (e.g. livestock and plants pathogens), or (d) if they were review studies or opinion pieces or editorials without primary data analysis. Additionally, due to the number of malaria research on the continent, malaria studies were excluded from this scoping review.  Studies were thematically grouped to examine various impacts of climate change in Sub-saharan Africa as identified in literature and further categorized into the following groups: highlighting research needs, informing science, providing solutions and knowledge to combat climate impacts, highlighting policy needs, and identifying resource needs. | 3 |
| Information sources | 6 | Specify all databases, registers, websites, organisations, reference lists and other sources searched or consulted to identify studies. Specify the date when each source was last searched or consulted.  Between October 30^th^, 2023, to 1^st^ August 2024, we used keyword search on Scopus, Embase, PubMed and Web of Science to identify studies included in table | 3 |
| Search strategy | 7 | Present the full search strategies for all databases, registers, and websites, including any filters and limits used.  We used keywords “climate change,” “health,”and “sub-Saharan Africa” and we filtered the publication date using a custom range of between January 1^st^, 2001, to August 1^st^, 2024. We also filtered for Article language to include only papers written in English language. | 3 |
| Selection process | 8 | Specify the methods used to decide whether a study met the inclusion criteria of the review, including how many  reviewers screened each record and each report retrieved, whether they worked independently, and if applicable, details of automation tools used in the process.  All papers were first assessed to see if it was published between January 1, 2001, and August 1, 2024. After this criterion is met, we proceeded to read the abstracts, to see if it conceptualizes climate change in Sub-saharan Africa explored the impacts of climate change and relevant to Africa and its health outcomes. Two reviewers screened each paper independently using covidence software to assess if they met our inclusion criteria. In cases where there was disagreement on the papers to be included, both reviewers were joined by another reviewer to. discuss why the papers should be included or excluded until a consensus was reached by two or all the reviewers for the paper to be included. Then, papers were read thoroughly to ensure that they were within the scope of the review. | 3 |
| Data collection process | 9 | Specify the methods used to collect data from reports, including how many reviewers collected data from each report, whether they worked independently, any processes for obtaining or confirming data from study investigators, and if applicable, details of automation tools used in the process.  All papers were imported into a google spreadsheet with the authors information, year of publication, journals used for publication and the studies IDs (DOIs and PMCID). Two reviewers worked independently to color code each paper into our thematic groups of climate change impacts that is relevant to the African region using each study title. Papers were thematically coded using guiding questions including country, climate impacts, health impacts, adaptation strategies and climate change mitigation strategies. | 4 |
| Data items | 10a | List and define all outcomes for which data were sought. Specify whether all results that were compatible with each outcome domain in each study were sought (e.g. for all measures, time points, analyses), and if not, the methods used  to decide which results to collect.  Journal articles were sought for relevant climate change effects such as water quality, flooding, drought on health in Sub-Sahara Africa. Results from previous studies focused on different health outcomes associated with changing climate in Africa. Some of these health outcomes includes.   - Water, sanitation, and hygiene issues (n=57) - Food security and malnutrition (n=40) - Physical illness (n=32) - Health risk associated with other pathogens (n=26) - Loss of livelihood (n=8) - Climate induced displacement (n=6) - Mental health (n=13) - Social determinant of health (n=7) - Gender based violence (n=2) - HIV/AIDs (n=2)   We examined where climate change and health research originated from in Sub-Sahara Africa to gain better insight into the research focus within the region and the geographical gap and in climate change and health studies within the region.   - Most of the studies were conducted in the South and East Africa region with (24) and (22) studies originating from South Africa and Kenya respectively, followed by Ghana (14), Tanzania (10) and Burkina Faso (9). - Twenty-one (26) studies included in this study were large scale studies conducted across multiple SSA countries. - Only 24 out of 53 countries in sub-Saharan Africa have published studies on climate change and health over the past 24 years and 15 countries had less than 5 published studies on climate change and health. - Majority of the studies published on climate change and health in sub-Saharan Africa between 2001 and August 2024 focused on extreme heat (71 studies), extreme precipitation events (52 studies), drought (45 studies) and flooding (34 studies). - A moderate number of studies have focused on infectious diseases (23 studies) and microbial water quality (23 studies). - Air quality (10 studies), chemical water quality (8) and natural disasters (8) have been understudied and researched in Sub-saharan Africa between 2001 and August 2024.   We also collected information on country, funding source, vulnerable groups identified, climate mitigation strategies and adaptation strategies. | 8 |
|  | 10b | List and define all other variables for which data were sought (e.g. participant and intervention characteristics, funding sources). Describe any assumptions made about any missing or unclear information.   - 20 studies highlighted research needs, 58 informed sciences (provides insight for climate trends and impacts), 43 provides solutions and knowledge to combat climate impacts, 15 highlights policy needs, 6 highlights resource needs and 11 studies explored multiples themes. - Of the studies considered, 45% (n=69) were funded by external grants, while 55% (n=84) were self-funded by researchers, declared as   “No external funding” in research publication. We assumed that when “no external funding was stated, the funding was from the researchers/institution”. | 6-9 |
| Study risk of bias assessment | 11 | Specify the methods used to assess risk of bias in the included studies, including details of the tool(s) used, how many reviewers assessed each study and whether they worked independently, and if applicable, details of automation tools used in the process.  Each study was closely read by two reviewers independently using covidence software to streamline and facilitate the review process. Where there are discrepancies on articles to be included, a third reviewer was invited to join a group review and thorough discussion of the study was done until there was a consensus. | 4 |
| Effect measures | 12 | Specify for each outcome the effect measure(s) (e.g. risk ratio, mean difference) used in the synthesis or presentation of results.  Not applicable |  |
| Synthesis methods | 13a | Describe the processes used to decide which studies were eligible for each synthesis (e.g. tabulating the study intervention characteristics and comparing against the planned groups for each synthesis (item #5)).  This was done by thematically grouping relevant health outcomes and based on these outcomes, x number of studies were grouped into the various themes after reading exhaustively and studying the main topic of discussion in the studies. Additionally, we wanted to see what the geographic distribution of studies were. Therefore, we used a map to represent this data. No advanced statistical analysis was performed. Data was presented using tables, charts and maps. | 3-4 |
|  | 13b | Describe any methods required to prepare the data for presentation or synthesis, such as handling of missing summary statistics, or data conversions.  Data was presented using tables, charts, and maps. Any article suggested but, which could not be obtained was excluded. | 3 |
|  | 13c | Describe any methods used to tabulate or visually display results of individual studies and syntheses.  Microsoft Excel was used to tabulate and display Pie charts and to visually display the heatmap of Africa where studies on climate change and health has been conducted. Charts were also made using Excel. Tables showing themes, number of studies in individual themes and citation of those studies were also included. | 5-9 |
|  | 13d | Describe any methods used to synthesize results and provide a rationale for the choice(s). If meta-analysis was performed, describe the model(s), method(s) to identify the presence and extent of statistical heterogeneity, and software package(s) used.  We synthesized our results by providing narratives which involves summarizing individuals study, identifying trends and drawing conclusions. The rationale behind this was to identify patterns and themes across each study for effective and broader understanding of the evidence. | 5-10 |
|  | 13e | Describe any methods used to explore possible causes of heterogeneity among study results (e.g. subgroup analysis, meta-regression).  Not applicable. |  |
|  | 13f | Describe any sensitivity analyses conducted to assess robustness of the synthesized results.  Not applicable |  |
| Reporting bias assessment | 14 | Describe any methods used to assess risk of bias due to missing results in a synthesis (arising from reporting biases).  We carried out multiple level of review and we had two or more reviewers reviewing independently to eliminate bias. We also did multiple level screening, and ensured that the screening was independent, but all the selected studies were agreed upon. | 3 |
| Certainty assessment | 15 | Describe any methods used to assess certainty (or confidence) in the body of evidence for an outcome.  We carried out multiple level of review and we had two or more reviewers reviewing independently to eliminate bias. | 3 |
| **RESULTS** | | |  |
| Study selection | 16a | Describe the results of the search and selection process, from the number of records identified in the search to the number of studies included in the review, ideally using a flow diagram.  Using keyword search in Scopus, Embase, PubMed, and Web of Science, 7851 studies were identified, of the 7851 identified studies, 921 were duplicates, and 6628 were excluded because they did not meet the inclusion criteria. A total of 302 studies were screened for eligibility. Of the 302 studies, 149 were excluded; 53 were reviews, 2 were specific to animal health, 44 were specific to malaria research, 23 were opinion pieces and correspondence, and 27 others had wrong geographical settings, study designs or outcomes. In total, 153 studies were included for review. See flow diagram below.  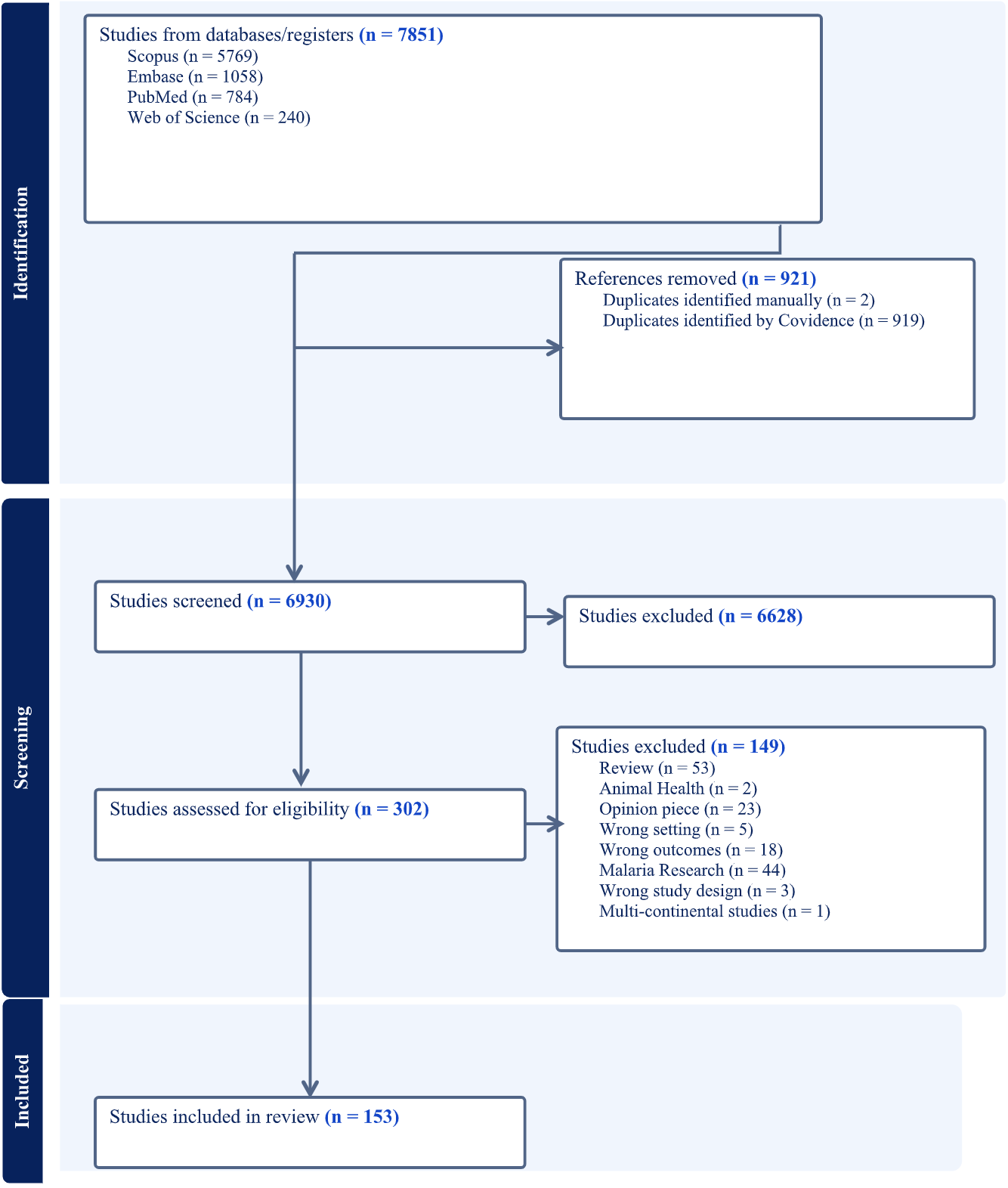 | 6 |
|  | 16b | Cite studies that might appear to meet the inclusion criteria, but which were excluded, and explain why they were excluded.  Studies on malaria were excluded because a lot of studies have been conducted in that area in SSA. Studies on animal health were excluded because our focus is on human health. Global studies were excluded because we want studies originated in SSA and unique to the impacts of climate change experienced in Africa. Review, opinion piece/editorial and book chapters were excluded because we wanted to focus our review using only original research. Studies with wrong settings within African continent that are not geographically considered as Sub-saharan Africa were also excluded. | 3,5 |
| Study characteristics | 17 | Cite each included study and present its characteristics.   \| **Climate Impact** \| **n** \| **Studies** \| \| --- \| --- \| --- \| \| Extreme Heat \| 71^*^ \| [4,10,11,14,18,19,31–95] \| \| Extreme Precipitation \| 52 \| [4,14,18,19,32–36,38–41,46–50,52,54,57,64,68,69,77–79,81,83,86,90,91,93–112] \| \| Drought \| 45 \| [2,14,34,36–49,97–103,113–132] \| \| Flooding \| 34 \| [2,2,4,8,14,19,35,38,40–48,52,56,57,67,68,90,95,97,99–101,103,107,128,133–136] \| \| Microbial Water quality \| 23 \| [20,35,61,81,95,105,106,108,135,137–151] \| \| Infectious Diseases \| 23 \| [1,11,14,47,60,61,91,102,108,138,143,151–161] \| \| Air Quality \| 10 \| [31–35,96,113,162–164] \| \| Chemical Water quality \| 8 \| [35,137,139,141,142,145,146,165] \| \| Natural Disasters \| 8 \| [8,16,41,42,47,67,94,100] \| | 5 |
| Risk of bias in studies | 18 | Present assessments of risk of bias for each included study.  We carried out multiple level of review and we had two or more reviewers reviewing independently to eliminate bias. | 3-4 |
| Results of individual studies | 19 | For all outcomes, present, for each study: (a) summary statistics for each group (where appropriate) and (b) an effect estimates and its precision (e.g. confidence/credible interval), ideally using structured tables or plots.  Not applicable |  |
| Results of syntheses | 20a | For each synthesis, briefly summarise the characteristics and risk of bias among contributing studies.  Not applicable |  |
|  | 20b | Present results of all statistical syntheses conducted. If meta-analysis was done, present for each the summary estimate and its precision (e.g. confidence/credible interval) and measures of statistical heterogeneity. If comparing groups, describe the direction of the effect.  Not applicable |  |
|  | 20c | Present results of all investigations of possible causes of heterogeneity among study results.  Not applicable |  |
|  | 20d | Present results of all sensitivity analyses conducted to assess the robustness of the synthesized results.  Not applicable |  |
| Reporting biases | 21 | Present assessments of risk of bias due to missing results (arising from reporting biases) for each synthesis assessed.  We carried out multiple level of review and we had two or more reviewers reviewing independently to eliminate bias. | 3-4 |
| Certainty of evidence | 22 | Present assessments of certainty (or confidence) in the body of evidence for each outcome assessed.  We carried out multiple level of review and we had two or more reviewers reviewing independently to eliminate bias. | 3-4 |
| **DISCUSSION** | | |  |
| Discussion | 23a | Provide a general interpretation of the results in the context of other evidence.  The results of this review support evidence that SSA is suffering and most likely, will continue to suffer from the effect of climate change more. Adversely compared to other global regions despite its insignificant contribution to greenhouse gas emission due to high vulnerability and low adaptive capacity. Drought, extreme heat, and infectious diseases such as cholera and diarrhea because of microbial and chemical contamination due to effects of climate change on water quality and access are few of the climate change effects present in the region. Most of the studies in this review focused on WASH issues, food security, physical illnesses such as heat exhaustion and respiratory distress from air pollution, and pathogen infections such as cholera, food security and malnutrition. However, the social, emotional, and mental aspects of climate change have been relatively understudied. Loss of livelihood due to the impact of floods, extreme temperature and drought on agricultural, resource-based occupation and outdoor informal economies has been reported in a few studies in the sub-Saharan African context.  The review also highlighted the geographic disparities exist in climate change and health research, with some countries being more studied and others lacking sufficient data. This disparity can lead to an incomplete understanding of climate change impacts across the entire sub-Saharan region. Even in countries where previous research has been conducted, previous authors have acknowledged the need for more primary climate data to promote data-driven decision making. | 10-14 |
|  | 23b | Discuss any limitations of the evidence included in the review.  Articles were selected from 2001 until August 2024. There is a possibility that more articles have been published on climate change and health in the region since then, and older studies might have important climate change and health findings that have been excluded from the current review. Additionally, while papers with a global focus might provide interesting insights, these papers were excluded because they do not provide the level of detail, and the local focus required to address climate change. Additionally, malaria research was excluded, therefore, this study does not address how climate change will affect the rate of malaria incidence in the future. Also, this review focuses on sub-Saharan Africa only,  Despite a comprehensive search strategy, some relevant studies might have been missed, particularly those published in non-indexed journals or in languages other than English. | 3 |
|  | 23c | Discuss any limitations of the review processes used.  The thematic synthesis approach might have introduced interpretive bias, where the review team’s perspectives influenced the identification of themes and patterns. Additionally, restricting the review to studies published in English could result in language bias, potentially excluding important findings published in other languages. | 3-4 |
|  | 23d | Discuss implications of the results for practice, policy, and future research.  This review revealed existing gaps and opportunities in climate research in SSA. First, geographic disparities exist, with some countries being more studied and others lacking sufficient data. There is insufficient information about climate impacts, vulnerability, and health impacts of climate change in countries without climate research. Even in countries where previous research has been conducted, previous authors have acknowledged the need for more primary climate data to promote data-driven decision making. Expanding research efforts to include understudied countries can provide a more comprehensive understanding of climate change impacts across SSA, ensuring that attention is given to those most impacted by climate change.  Many studies have not adequately considered the vulnerability of different population groups with an intersectional lens. There is a need for more comprehensive research focusing on community heterogeneity and intersectionality among the most affected groups. Furthermore, physical impact of climate change has been relatively well studied in certain countries of sub-Saharan Africa, there is a need for more research on the interconnections between climate change and socioeconomic wellness indicators. Additionally, while some climate themes have been extensively studied, others such as air pollution are less studied and require further attention because, climate change is expected to increase drought and air pollution in some regions of the world, including SSA.  In this study, more than half of the studies reviewed were self-funded by researchers. The large amount of self-funded research highlights the financial constraints faced by researchers, which can limit the scope, scale, and depth of their studies, ultimately hindering the development of comprehensive climate change mitigation and adaptation strategies. Therefore, there is an urgent need for increased investments in climate research. Despite increasing recognition of climate change impacts, the region remains underresearched, especially in areas beyond extreme heat, drought, precipitation events, air quality, and flooding. This has left substantial gaps in understanding the full spectrum of climate-induced health outcomes and the intersectional vulnerabilities that various populations face.  Future research should explore the interconnections between climate change and social determinants of health, including work, education, income, and housing, to inform more comprehensive and effective adaptation strategies. | 11-14 |
| **OTHER INFORMATION** | | |  |
| Registration and protocol | 24a | Provide registration information for the review, including register name and registration number, or state that the review was not registered.  This review was not registered |  |
|  | 24b | Indicate where the review protocol can be accessed, or state that a protocol was not prepared. |  |
|  | 24c | Describe and explain any amendments to information provided at registration or in the protocol. |  |
| Support | 25 | Describe sources of financial or non-financial support for the review, and the role of the funders or sponsors in the review.  No external funding support | PLOS One |
| Competing interests | 26 | Declare any competing interests of review authors.  No known competing financial interests that could have appeared to influence the work reported in this paper. | PLOS One |
| Availability of data, code and other materials | 27 | Report which of the following are publicly available and where they can be found template data collection forms; data extracted from included studies; data used for all analyses; analytic code; any other materials used in the review.  Not applicable |  |

*From:*  Page MJ, McKenzie JE, Bossuyt PM, Boutron I, Hoffmann TC, Mulrow CD, et al. The PRISMA 2020 statement: an updated guideline for reporting systematic reviews. BMJ 2021;372:n71. doi: 10.1136/bmj.n71
