# Supplementary material for: Assessing Sub-Saharan Africa’s readiness to address the impact of climate change and health: A scoping review
Source: PLoS One. 2025 Nov 11;20(11):e0315482. doi: 10.1371/journal.pone.0315482 (PMC12604764; doi:10.1371/journal.pone.0315482)
Supplement: Attachment — Submitted filename: Responses to reviewers comments 07282025.docx [file pone.0315482.s006.docx]

1295 N. Martin Avenue

P.O. Box 245209

Tucson, AZ 85724-5209

Tel: (520) 626-6317

Fax: (520) 626-8716

[http://publichealth.arizona.edu](http://publichealth.arizona.edu/)

| Ms. Ref. No.: | PONE-D-24-45324 |
| --- | --- |
| Manuscript Title: | Assessing Sub-Saharan Africa's Readiness to Address the Impact of Climate Change and Health: A Scoping Review |
| Journal: | PLOS |

Dear Editor and Reviewers,

We are submitting a revision of the original manuscript: “Assessing Sub-Saharan Africa's Readiness to Address the Impact of Climate Change and Health: A Scoping Review”. Thank you for this opportunity to revise and submit a new version of the manuscript. The suggestions are insightful and have improved this manuscript. We are grateful for the thoughtful comments we received from the Editor and the two reviewers. We have taken all the comments into account.

Please, see the responses below.

Editor’s Comments:

When submitting your revision, we need you to address these additional requirements.

1.  Please ensure that your manuscript meets PLOS ONE's style requirements, including those for file naming. The PLOS ONE style templates can be found at

[https://journals.plos.org/plosone/s/file?id=wjVg/PLOSOne_formatting_sample_main_body.pdf](https://track.editorialmanager.com/CL0/https:%2F%2Fjournals.plos.org%2Fplosone%2Fs%2Ffile%3Fid=wjVg%2FPLOSOne_formatting_sample_main_body.pdf/1/010f01950032bf59-42a8829e-32a6-40c0-ab33-98ae91d55831-000000/kyLjEKW4j5er6o5ZYNIUklhtJtvEjg7hyzpVt6KKd8Q=198)  and

[https://journals.plos.org/plosone/s/file?id=ba62/PLOSOne_formatting_sample_title_authors_affiliations.pdf](https://track.editorialmanager.com/CL0/https:%2F%2Fjournals.plos.org%2Fplosone%2Fs%2Ffile%3Fid=ba62%2FPLOSOne_formatting_sample_title_authors_affiliations.pdf/1/010f01950032bf59-42a8829e-32a6-40c0-ab33-98ae91d55831-000000/_z2fcfNAJeKVCvzYzZYORgMcmdkY14RbeueH0K7Zi-4=198)

**Response:** The manuscript title page and the main body has been formatted accordingly.

2. We note that your Data Availability Statement is currently as follows: [All relevant data are within the manuscript and its Supporting Information files.]

Please confirm at this time whether or not your submission contains all raw data required to replicate the results of your study. Authors must share the “minimal data set” for their submission. PLOS defines the minimal data set to consist of the data required to replicate all study findings reported in the article, as well as related metadata and methods (<https://journals.plos.org/plosone/s/data-availability#loc-minimal-data-set-definition>).

For example, authors should submit the following data:

- The values behind the means, standard deviations and other measures reported;

- The values used to build graphs;

- The points extracted from images for analysis.

Authors do not need to submit their entire data set if only a portion of the data was used in the reported study.

If your submission does not contain these data, please either upload them as Supporting Information files or deposit them to a stable, public repository and provide us with the relevant URLs, DOIs, or accession numbers. For a list of recommended repositories, please see <https://journals.plos.org/plosone/s/recommended-repositories>.

If there are ethical or legal restrictions on sharing a de-identified data set, please explain them in detail (e.g., data contain potentially sensitive information, data are owned by a third-party organization, etc.) and who has imposed them (e.g., an ethics committee). Please also provide contact information for a data access committee, ethics committee, or other institutional body to which data requests may be sent. If data are owned by a third party, please indicate how others may request data access.

3. Please amend either the title on the online submission form (via Edit Submission) or the title in the manuscript so that they are identical.

4. We note that Figure 2 in your submission contain [map/satellite] images which may be copyrighted. All PLOS content is published under the Creative Commons Attribution License (CC BY 4.0), which means that the manuscript, images, and Supporting Information files will be freely available online, and any third party is permitted to access, download, copy, distribute, and use these materials in any way, even commercially, with proper attribution. For these reasons, we cannot publish previously copyrighted maps or satellite images created using proprietary data, such as Google software (Google Maps, Street View, and Earth). For more information, see our copyright guidelines: <http://journals.plos.org/plosone/s/licenses-and-copyright>.

We require you to either (1) present written permission from the copyright holder to publish these figures specifically under the CC BY 4.0 license, or (2) remove the figures from your submission:

**Response:** Thank you for your feedback. We have replaced this figure with an original figure produced by the authors.

We have addressed this concern by recreating the map entirely in R Studio using open-source packages and publicly available shapefiles. Specifically, we used the ggplot2, sf, and rnaturalearth packages to generate the new map. The base map was created using data from Natural Earth (public domain), which provides freely available, high-quality cartographic datasets. We then overlaid our study site information and relevant environmental data to produce a map that conveys the same analytical content as the previous version but now adheres to copyright and data-use standards. The revised map was included in the updated manuscript that was submitted on 4/30/2025. Here are the publicly available domains that were used:

Massicotte P, South A (2025). rnaturalearth: World Map Data from Natural Earth. R package version 1.1.0, <https://docs.ropensci.org/rnaturalearth/>.

Wickham H (2016). ggplot2: Elegant Graphics for Data Analysis. Springer-Verlag New York. ISBN 978-3-319-24277-4, [https://ggplot2.tidyverse.org](https://ggplot2.tidyverse.org/).

Pebesma E, Bivand R (2023). Spatial Data Science: With applications in R. Chapman and Hall/CRC. doi:10.1201/9780429459016, <https://r-spatial.org/book/>.

5. We notice that your supplementary figure and table are included in the manuscript file. Please remove them and upload them with the file type 'Supporting Information'. Please ensure that each Supporting Information file has a legend listed in the manuscript after the references list.

**Response:** Thank you for your suggestion. All supplemental information has been removed, and a legend has been added after the reference list.

6. Please remove your figures from within your manuscript file, leaving only the individual TIFF/EPS image files, uploaded separately. These will be automatically included in the reviewers’ PDF.

**Response:** All figures have been removed from manuscript and have been included as TIFF image files.

7. Please review your reference list to ensure that it is complete and correct. If you have cited papers that have been retracted, please include the rationale for doing so in the manuscript text, or remove these references and replace them with relevant current references. Any changes to the reference list should be mentioned in the rebuttal letter that accompanies your revised manuscript. If you need to cite a retracted article, indicate the article’s retracted status in the References list and also include a citation and full reference for the retraction notice.
[Note: HTML markup is below. Please do not edit.]

**Reviewer's Responses to Questions**

Comments to the Author

1. Is the manuscript technically sound, and do the data support the conclusions?

The manuscript must describe a technically sound piece of scientific research with data that supports the conclusions. Experiments must have been conducted rigorously, with appropriate controls, replication, and sample sizes. The conclusions must be drawn appropriately based on the data presented.

Reviewer #1: Partly

Reviewer #2: Yes

Authors Response: Thank you for a honest assessment of our manuscript.

2. Has the statistical analysis been performed appropriately and rigorously?

Reviewer #1: N/A

Reviewer #2: Yes

3. Have the authors made all data underlying the findings in their manuscript fully available?

Reviewer #1: Yes

Reviewer #2: Yes

4. Is the manuscript presented in an intelligible fashion and written in standard English?

PLOS ONE does not copyedit accepted manuscripts, so the language in submitted articles must be clear, correct, and unambiguous. Any typographical or grammatical errors should be corrected at revision, so please note any specific errors here.

Reviewer #1: Yes

Reviewer #2: Yes

**5. Review Comments to the Author**

Please use the space provided to explain your answers to the questions above. You may also include additional comments for the author, including concerns about dual publication, research ethics, or publication ethics. (Please upload your review as an attachment if it exceeds 20,000 characters)

Reviewer # 1

| Comment | Response |
| --- | --- |
| Thank you for the opportunity to review this manuscript. The authors conduct a timely scoping review of climate change and health research in Sub-Saharan Africa and present the gaps and recommendations for researchers and policy makers to address these. Abstract - Add the word “studies” to the 8 behind natural disasters | Thank you for your feedback. We have now added the word studies to the abstract. |
| Introduction  Curious about the use of the terms “developing countries”. There has been a lot of discourse around this language. Most articles now use low-income countries or regions. | Thank you for your feedback. We acknowledge that the term “developing” can be problematic and have now replaced this word with “low-income” throughout this manuscript. |
| Remove this sentence – it repeats the same information above “Some countries are already experiencing higher rates than normal from malnutrition, diarrhea cholera, and vector-borne diseases such as dengue (21) | This sentence has now been removed from the manuscript |
| In the Research Questions why in #3 are you restricting strategies to air and water quality, extreme heat, enhancing resilience and mitigating health impacts? I would suggest instead, “What practical solutions and community-based adaptation strategies can be developed and implemented to enhance resilience and mitigate health impacts of climate change ?” | Thank you for your suggestion. The research question has now been modified to remove high level specificity especially in this context.  Methods |
| - I would remove this: Climate change impacts relevant to sub-Saharan Africa include extreme heat, food security, microbial and chemical water quality, flooding, and drought. There are more impacts than this and the language is not consistent | This sentence has now been removed |
| - You should include reasoning for this “No white papers, gray literature, review papers, or other sources were included.”, and only those written in English. A lot of community, government, NGO and academic research on climate change is published as these types of documents. You can make the argument though that these types of documents are less accessible and may be less likely to be used in planning as result | The reasoning for only including peer-reviewed papers have now been included in the manuscript. |
| Why was 2001 considered your starting point? | Author's Response: Thank you for this question. We selected 2001 as our starting point because it marks the beginning of the third assessment cycle of the Intergovernmental Panel on Climate Change (IPCC), which significantly advanced the global discourse on climate change and its regional impacts. This period also aligns with a noticeable increase in peer-reviewed literature focused on climate-health linkages in sub-Saharan Africa, making it a logical starting point for capturing the evolution of research in this field. |
| - I would rephrase this “(a) addressed the conceptualization of climate change in sub-Saharan Africa” to “Explores the effects of climate change in sub-Saharan Africa” | Thank you for your suggestion. We have now revised the language in this text. |
| Why would wildfires not be relevant to the African context? This is not accurate: Africa’s lush tropical forests face a surprising threat: fire, | We agree that although wildfires are not as prominent in Africa as they are in the Americas, they still occur. Thus, this sentence has been revised. |
| Again zoonotic impacts of climate change are of large concern, better rationale for the exclusion of the malaria research is required | The rationale for removing malaria research has been added to the manuscript. |
| The description of thematic analysis needs to be expanded; it is unclear if the themes were selected a priori based on the literature, OR if they were developed based on the research included in the literature review. IF it was the latter, then the “themes” would be results and should not be presented in the methods. If they were selected PRIOR to analysis/extraction of the included articles, then the rationale for how these were selected is required. | A more detailed description of the thematic analysis have been included in the manuscript |
| No extraction of data from the articles is described only the screening. Was the extraction also conducted by 2 people, was it done simultaneously with the screening? Please add more details. - How was data stored/analysed/verified? | Information of extraction, storage, verification and analysis has now been added to the manuscript. |
| These should be re-ordered in terms of magnitude: The majority of the studies published on climate change and health in sub-Saharan Africa between 2001 and August 2024 focused on extreme heat (71 studies), drought (45 studies), extreme precipitation events (52 studies) and flooding (34 studies). | Thank you for your feedback. The items have been re-ordered in terms of magnitude. |
| You use studies and publications interchangeably, please choose one | Thank you for your feedback. We have revised the manuscript to stick to one. |
| - I find lines 244-250 confusing. The way it is phrased sounds like a comparison. I would rephrase as: Most studies included in this scoping review focused on water, sanitation and hygiene issues (n=57), food security and malnutrition (n=40), physical illness (n=32) and health risks associated with pathogens (n=26). The remaining 53 articles covered loss of livelihood due to natural disasters, climate induced displacement, mental health, gender-based violence, HIV and death (Table 3). | The language has now been revised to reduce confusion. |
| - “This scoping review also examined specific countries in sub-Saharan Africa where climate change and health research originated.” Did you mean originated or took place? Originated implies it was conceived and conducted locally which is a great aspect to asses but it does not appear you did this | This language has now been revised |
| - I would move this to the figure description: “The findings were visualized on a map of Africa, with color coding from 4 to 24 studies, where a dark hue represents countries with more research and a lighter hue indicates countries with fewer studies. Countries shown in gray represent those with no published studies on climate change and health between 2001 and August 2024 that fit our criteria.” | The figure description has now been revised |
| - I think there is a missed opportunity in the section on vulnerability on what makes these groups vulnerable and if there are any commonalities between these groups, particularly how policies should be leveraged for these groups. | We have included a discussion on vulnerable groups |
| - Capitalize Indigenous | This has been revised. |
| - Pie charts don’t provide much value, I would consider another format or remove the figure all together | All pie charts have been removed and replaced with figures that provide more details. |
| Add the reference in Table 1 in the row “study across multiple countries in SSA” to their respective countries and add a * to indicate it covers additional countries. | We thank you for your suggestion. Thank you for your suggestion. We acknowledge the importance of studies conducted across multiple countries in sub-Saharan Africa. However, we have chosen not to assign these studies to specific countries in Table 1. This decision aligns with our emphasis on place-based, local solutions. This is an approach we believe is critical for addressing the nuanced and context-specific nature of climate challenges. Multi-country studies often lack the granularity and localized insights necessary to inform effective, targeted interventions at the national or community level |
| - There are a number of issues with the tables and figures please verify the numbers: o The number of entries in the supplementary figure 1 don’t add up to 153 | This figure has been revised and the numbers now add up to 153 |
| There are more entries than studies in Table 1 “Table 1. Number of Studies on Climate Change and Health by Country in Sub-Saharan Africa | This table has been revised and the numbers now add up to 153. |
| Discussion - Throughout the manuscript extreme weather is associated with climate change, however those two are not equivalent. For example, much of SSA experiences seasonal drought/dry seasons and rainy seasons. But, studies on these relationships between weather and health can be used to inform projections and impacts of climate change. More nuance is needed in the discussion | Thank you for this important observation. We agree that extreme weather and climate change are not equivalent, and that greater nuance is needed in how we present these concepts. While our manuscript discusses health impacts in the context of climate change, we recognize that many weather events like seasonal droughts or heavy rains are part of long-standing climatic patterns in sub-Saharan Africa and not necessarily attributable to anthropogenic climate change. To address this, we have revised the discussion to better distinguish between extreme weather events and long-term climate change. We now clarify that while certain weather-health relationships are shaped by existing seasonal patterns, they can still provide valuable insights into how health systems might respond under projected climate change scenarios. This distinction strengthens our argument for using weather-health evidence as a foundation for understanding and preparing for climate-related health risks. |
| - The argument for regional data is poor. It is important but the discussion presented here doesn’t illustrate why. | We have revised the discussion to include why regional data is essential in this study. |
| - You continue presenting results in the discussion section. Please review and then build off the results and link to the broader literature. | We have revised the discussion section |
| - Unclear why the malaria papers are brought up in the discussion when they were excluded from the review. | We have revised the discussion section |
| - I find the authors are confusing environment with being synonymous, e.g., this is a significant finding, mainly since the Lancet Global Burden of Disease (2016) reported that 33% of the global burden of stroke is attributed to environmental factors such as air pollution and lead exposure (177). <<- does climate change cause air pollution, OR is climate change caused by air pollution? I think some of these directionality questions require some nuance. Lead exposure can increase with climate change, but is more indirect as a result of historical leaded gasolines and poor safety regulations on lead use in production. | Thank you for your comment. Climate change worsens air pollution and exacerbates poor health outcomes, in this case, respiratory and cardiovascular diseases. None causes the other. |
| - It is unclear how the recommendations were developed from the themes/results of this scoping review. | Thank you. Based on the interpretation of the results, we formed conclusions and recommendations. We revised this section to provide more clarity. |
| - Your scoping review is on SSA – why are you referring to research relevant to the continent? | We have removed words such as “continent” and “Africa” |
| **Reviewer # 2**   The authors have articulated with scientific rigour gaps in research on climate and health and provided for aligned recommendations as per the identified gaps in research and implementation. Minor revisions are however required under the following sections outlined below; |  |
| Abstract and introduction: Authors have contextualized the study well in the abstract and introduction however, the abstract does not mention the approach used in inclusion of articles “PRISMA” | PRISMA and the inclusion criteria have now been included in the abstract and  Methods |
| Rational for classifying articles as focused on infectious diseases and the exclusion of 44 other research as Malaria research; Consider adding supplemental file on the infectious diseases covered and if there is any recommendation that stands out to mitigate the effects of climate change on the disease. Additionally, Rationale for this exclusion to be expanded or further clarified; Malaria transmission is a potential health outcome that is affected by climate change/resilience | Thank you for your feedback. The rational for exclusion and inclusion has been expanded upon, and the reason for excluding malaria has been included in the manuscript |
| Figures & Tables Figure 2 Title is not explicit mentioning the main bias of including only English written publication thus areas of Francophone, Portuguese and/or Arabic may be incorrectly classified here as lacking in climate and heath research. | The Figure 2 title has been revised to include this area of potential bias. |
| Review of figure 3 to align with findings provided: No external funding n=83 as per line 275: Additionally, graph does not highlight all findings presented in discussion argument from line 283-287: Self-funded research and locally/domestically funded research has been highlighted as main finding | The figure 3 graph has been modified to include self-funded research, local, and internationally funded research. |
| Final recommendation on line 495 to show limitations of the study and this paucity/gaps in publications is in for those done in English across the continent | The limitations of including only publications written in English has now been included in the manuscript. |

Thank you,

Aminata P. Kilungo

Arizona’s First University – Since 1885
